# From fragrance wheel to functional genes: a multi-omics investigation into fragrance type formation in ornamental *Hedychium* flowers

**DOI:** 10.1093/hr/uhag063

**Published:** 2026-02-27

**Authors:** Yiwei Zhou, Fang Wang, Xue Wei, Meixin Xiong, Ting Gao, Qin Wang, Lan Wang, Yunyi Yu, Rangcai Yu, Yanping Fan

**Affiliations:** The Research Center for Ornamental Plants, College of Horticulture, South China Agricultural University, Guangzhou 510642, China; The Research Center for Ornamental Plants, College of Horticulture, South China Agricultural University, Guangzhou 510642, China; The Research Center for Ornamental Plants, College of Horticulture, South China Agricultural University, Guangzhou 510642, China; The Research Center for Ornamental Plants, College of Horticulture, South China Agricultural University, Guangzhou 510642, China; The Research Center for Ornamental Plants, College of Horticulture, South China Agricultural University, Guangzhou 510642, China; The Research Center for Ornamental Plants, College of Horticulture, South China Agricultural University, Guangzhou 510642, China; The Research Center for Ornamental Plants, College of Horticulture, South China Agricultural University, Guangzhou 510642, China; The Research Center for Ornamental Plants, College of Horticulture, South China Agricultural University, Guangzhou 510642, China; College of Life Sciences, South China Agricultural University, Guangzhou 510642, China; The Research Center for Ornamental Plants, College of Horticulture, South China Agricultural University, Guangzhou 510642, China

## Abstract

Floral scent is a crucial quality trait in ornamental plants, yet research has been hampered by the lack of standardized sensory evaluation and the disconnect between genes, volatile compounds, and human perception. *Hedychium* is an excellent model for fragrance research due to its diverse fragrance types and rich volatile organic compound (VOC) profiles. This study establishes a sensory-omics framework to connect genetic pathways, VOC chemistry, and fragrance perception in *Hedychium* flowers. A multidisciplinary approach combined sensory panel analysis (developing a fragrance wheel), VOC profiling (HS–SPME–GC–MS and PTR–ToF–MS), transcriptomics, and functional characterization of key biosynthetic genes in 30 *Hedychium* accessions representing six fragrance types. Six distinct fragrance types were classified (e.g. strong floral, fruity), linked to specific VOC profiles (e.g. monoterpenoids, esters). PTR–ToF–MS validated rapid detection of key fragrance markers. Supervised partial least squares-discriminant analysis (PLS-DA) modeling of VOC signatures enabled fragrance-type classification and key variable selection. Transcriptomic analysis coupled with weighted gene co-expression network analysis (WGCNA) revealed two key gene modules—MEbrown (terpenoid-associated) and MEyellow (phenylpropanoid-associated)—that underlie fragrance variation. Functional validation through *in vitro* enzymatic assays and transient overexpression in tobacco leaves confirmed HcTPS1 as a eucalyptol synthase and HmBEAT1 as a benzyl acetate synthase. Collectively, these findings provide a comprehensive framework for *Hedychium* flowers, thereby elucidating the molecular and chemical basis of their sensory fragrance variation. The study delivers valuable genetic resources and a predictive model that establishes a foundation for the targeted breeding of floral fragrance in ornamental horticulture.

## Introduction

Floral scent plays an indispensable role in historical culture, social humanities, industrial applications, and natural ecosystems [1]. Plants of the same species may produce distinct fragrance profiles, which are composed of diverse volatile organic compounds (VOCs) including terpenoids, alcohols, aldehydes, esters, phenols, ketones, ethers, and aromatic compounds [2, 3]. These compounds are characterized by low molecular weight, low boiling point, and high volatility. Beyond their physiological and ecological roles in plants, they also carry substantial commercial value, finding applications in pharmaceuticals, cosmetics, food additives, pesticides, and other fields, where they contribute to ecological balance, human health, industrial utility, and biodiversity conservation [4, 5]. The combinatorial diversity of floral VOCs generates a wide spectrum of fragrance types that enhance the aesthetic value of ornamental plants while offering potential health benefits, such as mood regulation, sleep improvement, and pain relief [6].

Analogous to flower color classification into white, yellow, orange, and red spectra, floral fragrances can be categorized into distinct olfactory types that differentially affect human perception. For instance, roses exhibit tea, lemon, melon, and anise notes [7], while lilies demonstrate weak, cool, fruity, musky, honeyed, and characteristic lily fragrances [8]. Similar classifications have been reported for *Anthurium* [9], carnation [10], and tulip [11]. However, unlike color which can be easily categorized, the complexity of floral fragrances has limited sensory analysis to descriptive approaches or basic VOC correlations. Systematic evaluation methods are still lacking. While flavor wheel systems have been successfully developed for food products including beer [12], chocolate [13], baijiu [14], tea [15–17], human milk [18], brewing malt [19], and hot pot seasoning [20], such frameworks remain notably absent for floral fragrance evaluation. This absence significantly limits fragrance assessment.

The determinants of sensory fragrance types are multifaceted. Typically, different molecular structures yield distinct olfactory profiles, but similar perceptions can sometimes be triggered by dissimilar compounds [21]. Moreover, VOC quantification methods significantly influence correlation analyses [22]. This is demonstrated by the differential associations between lily fragrance preference and VOC measurements using normalization versus internal standard methods [23]. This raises critical questions: Can instrumental analysis replace human sensory evaluation to improve the objectivity and efficiency of fragrance classification? Successful precedents exist, including the identification of 15 tomato VOCs predictive of typical/non-typical aroma [24] and positive correlations between blueberry consumer preference with hexanal and 2-undecanone [25]. These findings underscore the necessity of considering both compound sensory attributes and quantification methods when developing instrument-based fragrance prediction models.

Despite decades of research employing multi-omics and molecular biology approaches to identify floral fragrance biosynthesis genes (e.g. *TPS*) and transcription factors in key ornamental species, including rose [26, 27], lily [8, 28–30], *Jasminum sambac* [31], and *Chimonanthus praecox* [32], the sensory impact of VOCs on human olfaction remains understudied. This knowledge gap has confined most floral research to ‘key gene-to-VOC’ associations, neglecting the critical ‘gene–VOC–sensory perception’ axis, thereby limiting targeted fragrance improvement and novel fragrance development. Addressing this limitation requires an interdisciplinary framework integrating sensory science, metabolomics, statistical modeling, bioinformatics, and molecular biology to systematically dissect the mechanisms underlying floral fragrance diversity.

*Hedychium* species are particularly suitable for sensory fragrance research, with our preliminary studies identifying a diverse array of VOCs across varieties with distinct fragrance profiles. Using this diverse germplasm, we will: (i) establish a floral fragrance evaluation wheel to characterize representative fragrance types; (ii) integrate sensory analysis with HS–SPME–GC–MS, PTR–ToF–MS, transcriptomics, and molecular biology; and (iii) employ multivariate statistics to elucidate the molecular basis of sensory variation. This systematic approach will establish a comprehensive floral fragrance research framework encompassing sensory evaluation, compound detection, key component identification, and gene functional analysis, providing valuable insights for understanding fragrance formation mechanisms and facilitating targeted genetic improvement.

## Results

### Sensory evaluation and fragrance wheel development

**Figure 1:**
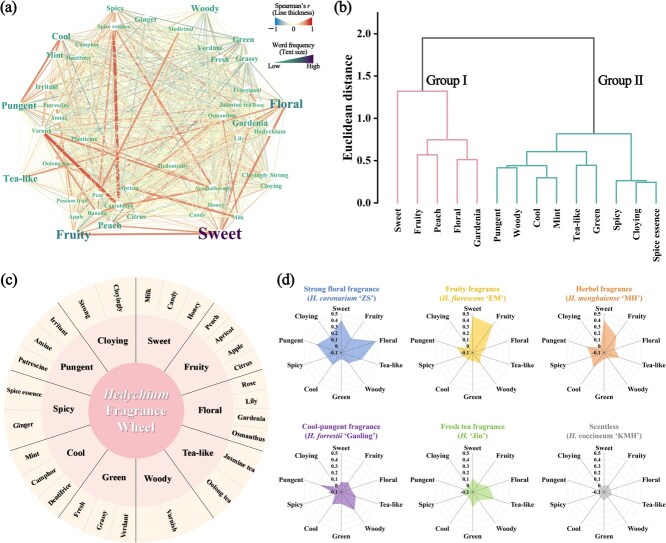
Construction of the sensory evaluation wheel for *Hedychium* fragrance and selection of representative fragrance types. (a) Correlation network of 46 sensory descriptors. (b) HCA of 14 key sensory descriptors. (c) Preliminary sensory evaluation wheel for *Hedychium* fragrance. (d) Six representative *Hedychium* fragrance types and their corresponding plant materials.

The plant materials used in this study are listed in Table S1. Sensory evaluation of *Hedychium* fragrance yielded 46 descriptive terms, which were collectively cited 1072 times. Among these, 13 terms—‘sweet’, ‘fruity’, ‘floral’, ‘tea-like’, ‘pungent’, ‘cool’, ‘woody’, ‘peach’, ‘gardenia’, ‘mint’, ‘green’, ‘grassy’, and ‘fresh’—were mentioned more than 30 times, indicating their significance as primary descriptors. Spearman correlation analysis revealed varying degrees of association among all 46 terms (Fig. 1a). Eleven terms with a geometric mean (*M*) value greater than 0.05 were selected for further analysis: ‘sweet’, ‘fruity’, ‘floral’, ‘pungent’, ‘tea-like’, ‘cool’, ‘peach’, ‘gardenia’, ‘woody’, ‘mint’, and ‘green’ (Table S2). Additionally, three supplementary terms—‘spicy’, ‘cloying’, and ‘spice essence’—were included based on expert panel discussions and discriminative relevance.

Hierarchical clustering analysis at a distance threshold of 1.5 grouped the 14 terms into two groups: Group I (‘sweet’, ‘fruity’, ‘peach’, ‘floral’, and ‘gardenia’) and Group II (‘pungent’ ,‘woody’, ‘cool’, ‘mint’, ‘tea-like’, ‘green’, ‘spicy’, ‘cloying’, and ‘spice essence’) (Fig. 1b). Subclusters merged semantically similar terms, including ‘fruity’ and ‘peach’, ‘floral’ and ‘gardenia’, ‘cool’ and ‘mint’, and ‘cloying’ and ‘spice essence’. The final fragrance wheel consisted of 10 primary descriptors (‘sweet’, ‘fruity’, ‘floral’, ‘tea-like’, ‘woody’, ‘green’, ‘cool’, ‘spicy’, ‘pungent’, and ‘cloying’) along with 27 secondary terms (Fig. 1c). Based on discriminative capacity and panel consensus, six principal fragrance types were identified, each represented by a distinct cultivar: strong floral (*Hedychium coronarium* ‘ZS’), fruity (*Hedychium flavescens* ‘EM’), herbal (*Hedychium menghaiense* ‘MH’), cool-pungent (*Hedychium forrestii* ‘Gaoling’), fresh-tea (*Hedychium* ‘Jin’), and scentless (*Hedychium coccineum* ‘KMH’) (Fig. 1d). To investigate common features of these fragrance types during peak bloom, 30 representative accessions (five per type) were selected for subsequent analysis based on sensory panel evaluation.

### VOC profiling of six *Hedychium* fragrance types using HS–SPME–GC–MS

**Figure 2:**
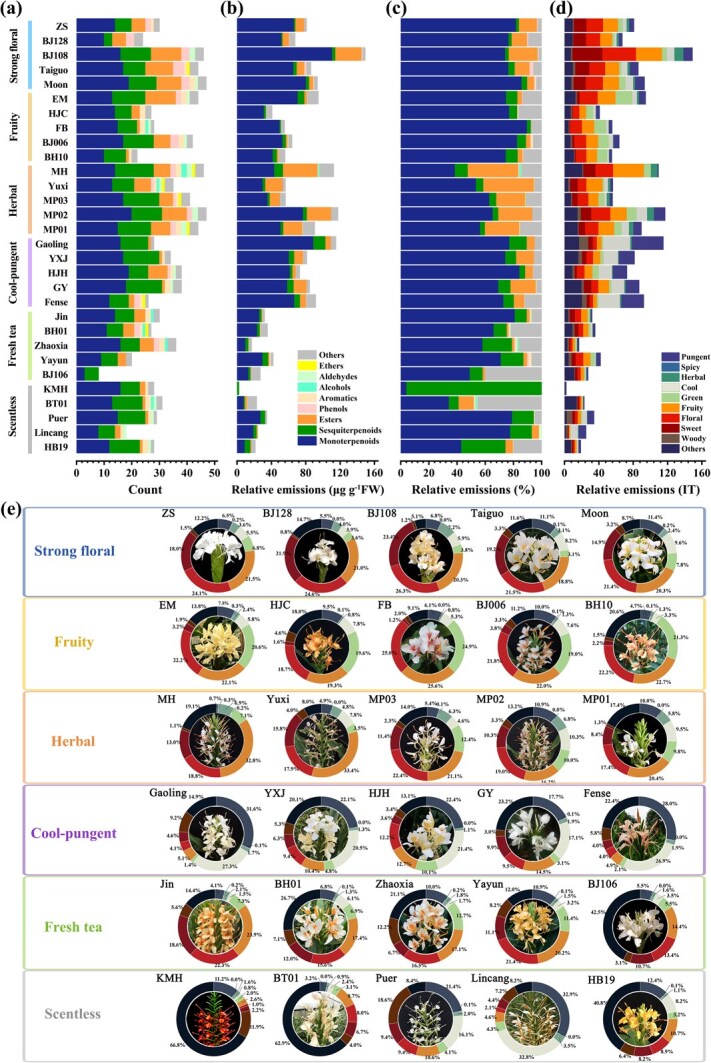
Floral volatile profiling of 30 representative *Hedychium* samples with six typical fragrance types based on HS–SPME–GC–MS. (a) Count statistics of different VOC classes. (b) Comparative analysis of relative VOC abundances across classes. (c) Proportional distribution of VOC classes by relative abundance. (d) Relative VOC abundances classified by incense tone. (e) Proportional distribution of VOC classes classified by incense tone.

The total ion chromatogram (TIC) profiles demonstrated both the overall similarity of floral fragrance characteristics within the same sensory fragrance type and the distinct differences between different fragrance types (Figs S1-S6). HS–SPME–GC–MS analysis identified 81 VOCs across 30 *Hedychium* accessions (Table S3; Fig. S7). The enumeration of volatile compounds revealed distinct quantitative patterns among *Hedychium* fragrance types. Monoterpenoids were numerically predominant in strong floral, fruity, herbal, and cool-pungent types, while fresh-tea and scentless types contained fewer monoterpenoids (Fig. 2a; Table S4). Sesquiterpenoids counts peaked in herbal types, were minimal in fresh-tea types, and showed intermediate variation in the remaining four fragrance types. Total VOC emissions were substantially higher in strong floral, fruity, herbal, and cool-pungent types compared to fresh-tea and scentless types (Fig. 2b; Table S5). Among all compound classes, monoterpenoids exhibited the highest emission levels across these four fragrant types, followed by esters which were particularly abundant in herbal and strong floral types (Fig. 2b). In terms of relative composition, monoterpenoids dominated in strong floral, fruity, cool-pungent, and fresh-tea types, while sesquiterpenoids prevailed in scentless types (Fig. 2c; Table S6). Esters showed highest relative abundance in herbal types, followed by strong floral types.

The incense tone of detected VOCs were classified into 10 categories (pungent, spicy, herbal, cool, green, fruity, floral, sweet, woody, and others) based on FlavorDB2 database [33]. The six *Hedychium* fragrance types exhibited significant differences in both the absolute concentrations and relative proportions of olfactory notes (Fig. 2d; Table S7). Strong floral types showed significantly higher levels of sweet and floral notes compared to other fragrance types, while fruity types were characterized by predominant green notes. Herbal types displayed distinctly elevated herbal characteristics, and cool-pungent types contained markedly higher concentrations of both pungent and cool notes. In terms of relative composition, strong floral types demonstrated the highest proportion of sweet notes, whereas green notes dominated in fruity types (Fig. 2e; Table S8). Herbal notes showed maximum representation in herbal types, and cool-pungent types were distinguished by the highest relative amounts of pungent and cool notes. Fresh tea types contained elevated proportions of green, floral, and fruity notes, while scentless types exhibited no consistent pattern in note distribution, which may be attributed to their extremely low overall volatile content (Fig. 2e; Table S8).

### PTR–ToF–MS analysis of six *Hedychium* fragrance types

**Figure 3:**
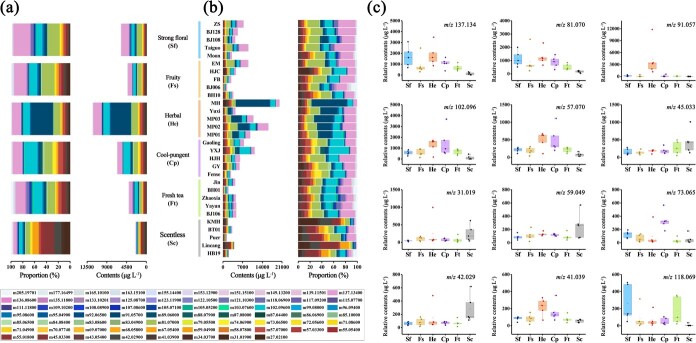
Floral volatile profiling of 30 representative *Hedychium* samples with six typical fragrance types based on PTR–ToF–MS. (a) Mean emission levels and proportions of 68 mass spectral peaks across six fragrance types. (b) Mean emission levels and proportions of 68 mass spectral peaks in 30 representative samples. (c) Top 12 mass spectral peaks with emissions exceeding 100 μg∙l^−1^.

The mass spectral profiles from PTR–ToF–MS analysis demonstrated distinct signal intensity variations across different sensory fragrance types (Figs S8-S13). The detection identified 68 mass peaks with characteristic abundance patterns among the fragrance categories (Table S9; Fig. S14). Total ion intensity followed the hierarchy: herbal > cool-pungent > strong floral > fruity > fresh-tea > scentless (Fig. 3a and b). Twelve peaks exceeded 100 μg l^−1^ average intensity, with *m*/*z* 137.134 (monoterpenes) showing highest abundance (Fig. 3c). This monoterpene marker exhibited maximal levels in strong floral and herbal types, intermediate levels in cool-pungent, fruity, and fresh-tea types, and minimal detection in scentless types. Other significant peaks included *m*/*z* 81.070, 91.057, 102.096, 57.070, 45.033, 31.019, 59.049, 73.065, 42.029, 41.039, and 118.069, collectively representing characteristic fragrance profiles across different fragrance types.

### Correlation analysis between VOCs of HS–SPME–GC–MS and mass peak of PTR–ToF–MS

Building upon our previous findings demonstrating concordance between HS–SPME–GC–MS and PTR–ToF–MS detection in *Hedychium* floral fragrance analysis [34], we performed PLS regression to further validate the representativeness of PTR–ToF–MS mass peaks for HS–SPME–GC–MS detected VOCs. The loading plot revealed substantial overlap between specific mass peaks and VOCs, indicating strong correspondence (Fig. S15a). Jackknife testing (*P* < 0.05) identified 35 mass peaks showing positive correlations with 34 VOCs, including 13 monoterpenoids, 7 sesquiterpenoids, 1 alcohol, 1 aldehyde, 6 esters, 1 aromatic, 1 ether, 2 phenols, and 1 other VOC (Fig. S15b and c; Table S10). These encompassed key fragrance compounds such as (*E*)-β-ocimene, linalool, eucalyptol, benzyl acetate, and methyl benzoate. Notably, (*E*)-β-ocimene showed significant correlation with *m*/*z* 96.094 and 107.086, while linalool correlated with *m*/*z* 68.058, 67.054, and 155.144 (the latter corresponding to linalool's molecular weight) (Fig. S15c). Eucalyptol associated with *m*/*z* 84.084 and 151.151, benzyl acetate with *m*/*z* 57.033 and 108.089, and methyl benzoate with *m*/*z* 57.033, 92.065, and 108.089. Several mass peaks demonstrated polyvalent correlations, with *m*/*z* 73.065, 74.069, and 96.094 showing significant associations with 7, 5, and 5 VOCs, respectively, suggesting fragment ion overlap among multiple compounds.

### Identification of fragrance-type markers and key determinants

**Figure 4:**
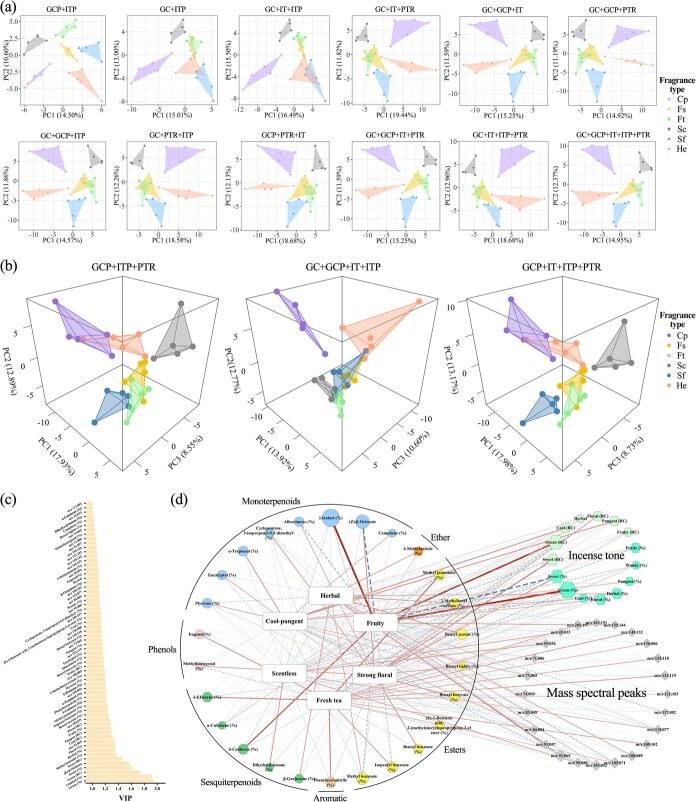
Identification of key variables driving *Hedychium* fragrance-type differentiation via PLS-DA. (a) Two-dimensional score plot of 12 datasets (*Q^2^* > 0.5). (b) Three-dimensional score plot of three datasets (*R^2^Y* > 0.83, *Q^2^* > 0.5, *RMSEE* < 0.2). (c) Important variables (VIP > 1) from the ‘GCP + IT+ITP + PTR’ dataset model. (d) Association network between 65 key variables (VIP > 1) and six fragrance types. VOC relative content (GC), VOC percentage (GCP), IT, ITP from HS–SPME–GC–MS, and mass peak intensity (PTR) from PTR–ToF–MS.

To identify potential discriminative markers for *Hedychium* fragrance types, we conducted multivariate analysis of five datasets: VOC relative content (GC), VOC percentage (GCP), incense tone relative content (IT), incense tone percentage (ITP) from HS–SPME–GC–MS, and mass peak intensity (PTR) from PTR–ToF–MS. Initial unsupervised principal component analysis (PCA) showed limited discriminative power, prompting supervised PLS-DA analysis of 31 combined datasets (Fig. S16). Model evaluation revealed three optimal datasets (GCP + ITP + PTR, GC + GCP + IT+ITP, and GCP + IT+ITP + PTR) with *R^2^Y* > 0.83, *Q^2^* > 0.5, and *RMSEE* < 0.2 (Fig. 4a and b; Table S11). The most informative dataset (GCP + IT + ITP + PTR) identified 65 variables with variable importance in projection (VIP) > 1, highlighting their discriminative potential (Fig. 4c; Table S12).

Network analysis associated specific variables with fragrance types: strong floral with sweet (%), sweet (RC), benzyl butyrate (%), (*E*)-β-ocimene, and benzyl benzoate (%); fruity with green (%) and linalool (%); herbal with benzyl acetate (%), floral (%), and 2-methylbutyl acetate (%); cool-pungent with cool (RC) and pungent (RC); fresh tea with methyl jasmonate (%), phenylacetonitrile (%), δ-elemene (%), and eugenol (%); and scentless with negative associations for fruity (%) and herbal (%) (Fig. 4d).

### Transcriptomic analysis

**Figure 5:**
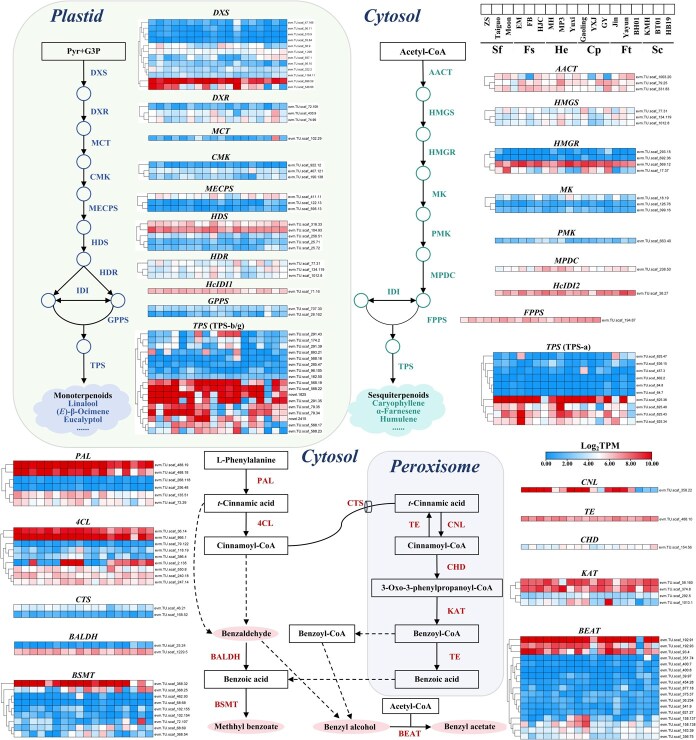
Comparative analysis of DEGs in VOCs biosynthetic related pathways across 18 representative samples of six fragrance types. Pyr, Pyruvate; G3P, glyceraldehyde-3-phosphate; DXR, 1-deoxy-d-xylulose-5-phosphate reductoisomerase; MCT, 2-C-methyl-d-erythritol 4-phosphate cytidylyltransferase; CMK, 4-(Cytidine 5′-diphospho)-2-C-methyl-d-erythritol kinase; MECPS, 2-C-methyl-d-erythritol 2,4-cyclodiphosphate synthase; HDR, 4-Hydroxy-3-methylbut-2-enyl diphosphate reductase; IDI, isopentenyl diphosphate isomerase; AACT, acetoacetyl-CoA thiolase; HMGS, 3-Hydroxy-3-methylglutaryl-CoA synthase; MK, mevalonate kinase; PMK, phosphomevalonate kinase; MDPK, mevalonate diphosphate decarboxylase; GPPS, geranyl diphosphate synthase; FPPS, farnesyl diphosphate synthase; C4H, cinnamate 4-hydroxylase; BALDH, benzaldehyde dehydrogenase; CFAT, coniferyl alcohol acyltransferase; CNL, cinnamoyl-CoA ligase; CHD, cinnamoyl-CoA hydratase/dehydrogenase; KAT, 3-Ketoacyl-CoA thiolase.

Quality assessment of 54 transcriptomes from 18 accessions yielded 2 773 812 758 raw reads (~416.1 Gb), with 2 383 938 010 clean reads (~354.6 Gb) retained after filtering (Table S13). Data quality metrics showed Q_20_ (95.80%–99.06%; mean, 97.84%) and Q_30_ (88.26%–95.72%, mean 93.33%). Correlation and PCA analyses confirmed data normality for downstream analysis (Figs S17 and S18). Focusing on key VOC biosynthesis pathways, we identified 33 MEP pathway DEGs, 17 MVA pathway DEGs, and 27 terpene synthases genes (10 *TPS*-*a*, 12 *TPS*-*b*, 5 *TPS*-*g*) in terpenoid biosynthesis, along with 52 phenylpropanoid pathway DEGs including 9 benzoic acid/salicylic acid carboxyl methyltransferase (*BSMTs)* and 17 benzylalcohol O-acetyltransferases (*BEATs)* (Fig. 5; Table S14). These findings provide molecular insights into the genetic basis of *Hedychium* fragrance diversity.

### Identification of key floral fragrance biosynthesis genes by WGCNA and PLS-DA

**Figure 6:**
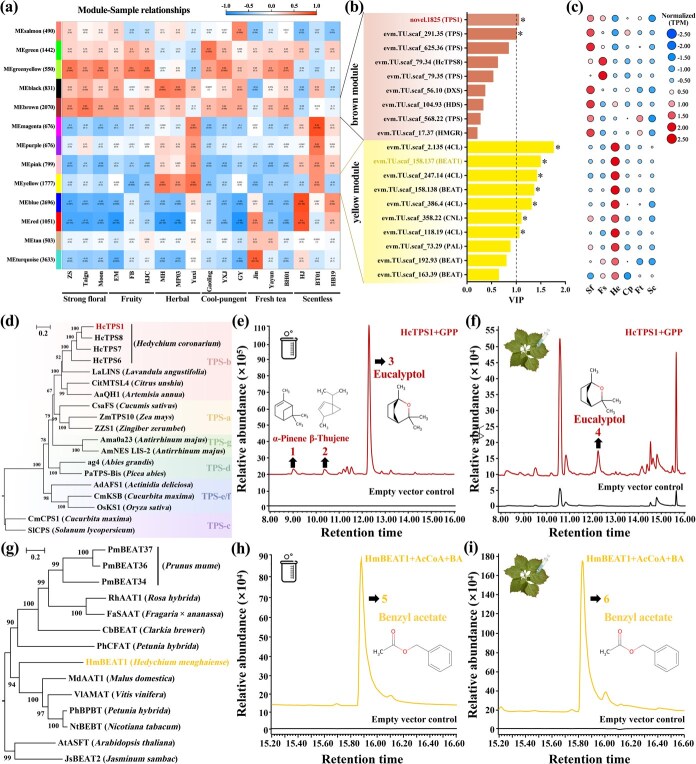
Functional characterization of key synthase genes influencing fragrance types and critical VOCs. (a) Heatmap of sample-module correlations from WGCNA (13 gene modules). (b) VIP scores of 19 key synthase genes from the MEbrown and MEyellow modules. Genes marked with an asterisk (*) indicate a VIP score greater than 1. (c) Relative expression of 19 synthase genes across fragrance-type samples. (d) Phylogenetic tree of HcTPS1/6/7/8 and other plant TPSs. (e) *In vitro* enzymatic assay products of HcTPS1 with GPP. Peaks 1–3 correspond to α-pinene, β-thujene, and eucalyptol, respectively. (f) *In planta* transient overexpression of *HcTPS1* in *Nicotiana benthamiana* leaves, showing altered eucalyptol emission. Peak 4 represents eucalyptol. (g) Phylogenetic tree of HmBEAT1 and other BAHD acyltransferase family enzymes. (h) *In vitro* enzymatic assay products of HmBEAT1 with acetyl-CoA and benzyl alcohol. (f) *In planta* transient overexpression of *HmBEAT1* in *N. benthamiana* leaves, demonstrating altered benzyl acetate emission. Peaks 5 and 6 represent benzyl acetate.

To identify crucial genes involved in the formation of characteristic fragrance compounds across different *Hedychium* fragrance types, we performed weighted gene co-expression network analysis (WGCNA). After filtering low-expressed genes, 17 194 genes were retained for analysis (Table S15). Soft threshold determination revealed optimal network topology at power = 5 (*R^2^* = 0.82), yielding 13 distinct gene modules through hierarchical clustering and merging procedures (Fig. 6a; Fig. S19). Correlation analyses between gene modules and both samples/key aromatic compounds identified two functionally significant modules: MEbrown (terpenoid-associated) and MEyellow (phenylpropanoid-associated) (Fig. 6a; Fig. S20).

The MEbrown module contained nine terpenoid biosynthesis genes, including six *TPSs*, one 1-deoxy-d-xylulose-5-phosphate synthase (*DXS*), one 4-hydroxy-3-methylbut-2-enyl diphosphate synthase (*HDS*), and one 3-hydroxy-3-methylglutaryl-CoA reductase (*HMGR*). Notably, evm.TU.scaf_79.34 (previously characterized as *HcTPS8*, a linalool synthase gene in our prior study) was recovered [35], validating the analytical approach. The MEyellow module comprised ten phenylpropanoid pathway genes: four *BEATs*, four 4-coumarate-CoA ligases (*4CLs*), one phenylalanine ammonia-lyase (*PAL*), and one coniferyl alcohol acyltransferase (*CNL*).

We then conducted partial least squares-discriminant analysis (PLS-DA) and calculated VIP scores to further prioritize candidate genes within these key modules. Genes with VIP scores >1.0, indicating a strong discriminatory power for fragrance types, were considered high-priority candidates. In the MEbrown module, two *TPS* genes (novel.1825 and evm.TU.scaf_291.35) met this criterion. In the MEyellow module, seven genes, including two BEATs (evm.TU.scaf_158.137 and evm.TU.scaf_158.138) and four 4CLs, were identified as high VIP candidates (Fig. 6b, c; Tables S16, S17). Among these, the two BEAT genes showed the most pronounced and specific upregulation in samples of the herbal fragrance type. TPS and BEAT enzymes are known to catalyze the final committed steps in the biosynthesis of terpenoid and benzenoid/phenylpropanoid volatiles (such as benzyl acetate), respectively. Therefore, to experimentally validate the enzymatic functions of candidate genes from the two key biosynthetic pathways, we selected the top-ranking TPS candidate from the MEbrown module (novel.1825, designated *TPS1*) and the BEAT gene with the highest VIP score and the strongest association with the Herbal fragrance type from the MEyellow module (evm.TU.scaf_158.137, designated *BEAT1*) for functional characterization (Fig. 6b).

### Cloning and functional characterization of *HcTPS1* and *HmBEAT1*

Full-length coding sequences of *HcTPS1* and *HmBEAT1*were cloned from petal cDNA of *H. coronarium* ‘ZS’ and *H. menghaiense* ‘MH’, respectively, which were the cultivars showing the highest expression of the corresponding genes. *HcTPS1* (1773 bp ORF) and *HmBEAT1* (1257 bp ORF) encode 591-aa (69.1 kDa) and 541-aa (46.0 kDa) proteins, respectively (Table S18). The primer sequences used for the relevant cloning and vector construction are listed in Table S19. Amino acid sequence alignment reveals that HcTPS1 and HmBEAT1 contain the conserved motifs DDXXD and HXXXD, respectively (Figs S21 and S22). Phylogenetic analysis positioned HcTPS1 within a clade containing known monoterpene synthases HcTPS6/7/8 (Fig. 6d) [35, 36]. Heterologous expression in *Escherichia coli* and enzymatic assays using geranyl diphosphate (GPP) as substrate demonstrated that HcTPS1 functions as a monoterpene synthase, primarily catalyzing the formation of eucalyptol with trace amounts of α-pinene and β-thujene (Fig. 6e). Transient overexpression of *HcTPS1* in tobacco leaves resulted in detectable emission of eucalyptol, which was absent in the control leaves (Fig. 6f). Phylogenetically, HmBEAT1 clustered with functionally characterized acyltransferases such as MdAAT1, VlAMAT, NtBEAT, and PhBPBT (Fig. 6g). In vitro enzymatic assays demonstrated that HmBEAT1 possesses benzyl alcohol acetyltransferase activity, catalyzing the formation of benzyl acetate from benzyl alcohol and acetyl-CoA (Fig. 6h). Furthermore, transient expression of *HmBEAT1* in tobacco leaves, supplemented with acetyl-CoA and benzyl alcohol, led to the release of benzyl acetate, which was not detected in control infiltrations (Fig. 6i). Taken together, these molecular and biochemical characterizations establish HcTPS1 and HmBEAT1 as key enzymes responsible for the biosynthesis of the monoterpenoid eucalyptol and the aromatic ester benzyl acetate, respectively, in *Hedychium* floral fragrance formation.

## Discussion

### A fragrance wheel for *Hedychium*: Bridging the standardization gap

While sensory analysis has been extensively studied in food science [37], its application in ornamental plant fragrance research remains limited. Although preliminary fragrance classifications exist for important ornamentals such as roses [7, 38], lilies [8], *Anthuriums* [9], carnations [10], and tulips [11], the absence of standardized sensory evaluation wheels has significantly hindered the scientific rigor and reproducibility of floral fragrance assessment. Our study addressed this gap by establishing a sensory panel that evaluated *Hedychium* fragrances, identifying 10 key descriptors through geometric mean and correlation analyses, and developing the first sensory evaluation wheel for ornamental plant fragrances. This wheel provides a unified and scientifically grounded standard for the sensory evaluation of *Hedychium* floral fragrances. Based on the evaluation wheel, six principal fragrance types were identified and categorized: strong floral, fruity, herbal, cool-pungent, fresh tea, and scentless.

The proposed fragrance wheel represents a foundational step toward standardizing sensory evaluation in *Hedychium*, though certain limitations must be acknowledged. First, its current form is based on evaluations of a specific set of cultivars at full bloom; future validation across developmental stages and environments is required. Second, the ‘scentless’ category warrants careful interpretation, as it may reflect either true biochemical absence or VOC emissions below human detection thresholds. While this distinction highlights the interplay between instrumental limits and perceptual thresholds, the category retains biological relevance. It captures an important consumer trait and, critically, serves as a valuable phenotypic control for comparative studies aimed at deciphering the genetic and metabolic basis of fragrance biosynthesis through omics approaches.

Despite these limitations, the methodological framework is designed for extensibility. The data-driven approach used to derive core descriptors provides a reproducible blueprint that can be applied to broader *Hedychium* diversity—likely refining categories and adding new descriptors—and adapted to other ornamental genera. Thus, this work not only provides a tool for *Hedychium* but also offers a transferable template for constructing sensory wheels in under-studied species, enhancing objectivity and comparability in floral fragrance research.

### Decoding fragrance types: Linking sensory perception to VOC fingerprints

A persistent challenge in floral fragrance research is the reliance on labor-intensive olfactory assessments, which demand extensive expert training. Recent advances in PTR–ToF–MS offer a promising alternative for rapid, high-throughput volatile phenotyping [39]. Studies on blueberry [40], peach [41], and star jasmine [42] demonstrate that integrating PTR–ToF–MS with GC–MS can establish robust VOCs evaluation systems. Building on our prior work [43], we identified consistent mass spectral signatures in *Hedychium*, including: (i) *m*/*z* 155.144 (linalool), (ii) *m*/*z* 57.033/108.089 (benzyl acetate), and (iii) *m*/*z* 151.151 (eucalyptol). These findings underscore PTR–ToF–MS's utility for rapid detection of key floral volatiles.

Understanding fragrance diversity formation requires consideration of multiple VOC characteristics and appropriate machine learning approaches to model complex data relationships [44, 45]. PLS-DA is a classical supervised classification model within the machine learning domain [46]. Our supervised PLS-DA analysis revealed the ‘GCP + IT + ITP + PTR’ dataset as most discriminative for the six fragrance types, identifying key explanatory features including: ‘Sweet’ and ‘benzyl benzoate’ for strong floral; ‘green’ and ‘linalool’ for fruity; ‘benzyl acetate’ for herbal; ‘cool’ and ‘pungent’ notes for cool-pungent types. These associations provide a foundation for targeted fragrance improvement and practical applications.

It is important to emphasize that the relationships identified between these key VOCs and sensory fragrance types represent statistical correlations derived from our analysis, not causal determinants. Sensory fragrance profiles are typically shaped by the synergistic interplay of multiple VOCs rather than by a single marker compound. The key markers identified through our correlation analysis were established within the specific context of the *Hedychium* fragrance types examined in this study. Notably, the sensory attributes of these key VOCs align with descriptions in established odor databases [33], and these attributes were largely consistent with the results of our independent sensory panel evaluation. To further elucidate the precise role these VOCs play in forming their corresponding sensory profiles, future studies employing more definitive approaches—such as in vitro scent reconstitution and omission experiments—are warranted.

Our integrative analysis reveals both a fundamental concordance and inherent complexities between sensory and instrumental data in fragrance profiling. The strong correlation between specific sensory fragrance type (e.g. herbal) and key volatile markers (e.g. benzyl acetate) underscores a qualitative agreement, providing a chemical basis for the fragrance types defined by our sensory wheel. This aligns with the established principle that odor perception is driven by key odorants, often present in trace amounts but with high odor activity values, rather than by the most abundant volatiles [47, 48]. However, discrepancies arise from the non-additive nature of olfactory perception. Complex synergistic or masking interactions within the VOC mixture mean the overall scent is not a mere sum of its parts, and human perception responds non-linearly to concentration changes [49]. Therefore, while instrumental profiling objectively quantifies volatile composition, sensory evaluation remains indispensable for capturing the holistic and perceptually relevant fragrance phenotype, explaining why the two approaches are complementary yet distinct.

### Gene modules and key enzymes: Molecular underpinnings of fragrance variation

The biosynthetic pathways of many floral volatiles are well-characterized [50]. For instance, the molecular mechanisms underlying floral scent have been extensively dissected in model ornamentals such as rose (*Rosa* spp.) and lily (*Lilium* spp.), providing valuable benchmarks for this study. In roses, the characteristic scent is dominated by monoterpene alcohols (e.g. geraniol, citronellol) and 2-phenylethanol, governed by dedicated *TPSs* [51], *CADs* [27], and *AAAT*/*PAR* [52, 53] genes. Similarly, in lilies, the balance between monoterpenoid and benzenoid/phenylpropanoid (e.g. methyl benzoate) volatiles defines major scent phenotypes, with recent studies highlighting the role of *TPSs* [28, 29] and *AAT1* [54] genes. While these foundational studies have successfully identified terminal synthase genes responsible for prominent volatile compounds through integrated chemical, molecular, and functional approaches, the collective contribution of these genes and their metabolic products to the integrated sensory perception of a holistic floral scent remains less comprehensively understood.

In this study, by integrating sensory classification with transcriptomics and multivariate statistics, we identified two critical gene modules: MEbrown (terpenoid-associated) containing six *TPS* genes including the previously characterized linalool synthase gene *HcTPS8* (evm.TU.scaf_79.34) [35], and MEyellow (phenylpropanoid-associated) with four *BEAT* genes. Functional characterization of top VIP scoring genes revealed *HcTPS1* as a eucalyptol synthase gene and *HmBEAT1* as a benzyl alcohol acetyltransferase—enzymes gene producing key volatiles for strong floral and herbal fragrance types, respectively. To our knowledge, this study is the first to identify both benzyl acetate and its key biosynthetic gene in the genus *Hedychium*, although this floral compound and its corresponding synthase gene have been reported in other plants, such as *Clarkia breweri* [55, 56], *Prunus mume* [57], and jasmine [58]. However, compared to terpenoids and TPS genes, research on benzyl acetate and its associated BEAT genes remains less comprehensive, particularly regarding their evolutionary diversity across plants, warranting further investigation. These findings expand our understanding of *Hedychium* fragrance diversity while highlighting the need to characterize additional regulatory genes to complete the molecular model of fragrance type determination.

The functional context of these genes in *Hedychium* reveals lineage-specific diversification. While BEAT homologs have been functionally characterized in several species, their role in shaping sensory perceptions rarely dissected in the context of a defined sensory wheel. Our integrated analysis reveals that *HmBEAT1* is a key determinant for the ‘herbal’ sensory type. This contrasts with the ecological context of benzyl acetate production in, for example, *C. breweri*, where it is part of a strong, sweet scent syndrome adapted to moth pollination [55, 56]. Therefore, HmBEAT1 exemplifies how conserved biosynthetic capacity (acetyl transfer to benzyl alcohol) can be recruited to contribute to novel scent phenotypes through regulatory or metabolic network evolution.

It is noteworthy that both key gene modules—the terpenoid-associated MEbrown module and the phenylpropanoid-associated MEyellow module—contained additional genes within the same pathways as *HcTPS1* and *HmBEAT1*, respectively. Interestingly, the co-expressed *HcTPS1* (eucalyptol synthase) and *HcTPS8* (linalool synthase) [35] possess distinct catalytic activities, suggesting the other co-expressed TPSs may be responsible for synthesizing other important monoterpenes. Regarding the other BEATs co-expressed with *HmBEAT1*, we postulate they may serve functionally redundant roles, as they are specifically expressed in petals of the three samples classified as herbal fragrance type, and benzyl acetate (the product of HmBEAT1) is a characteristic VOC for this fragrance type. Furthermore, genes within the same biosynthetic pathway are often co-regulated [5]. The other *TPSs* co-expressed with *HcTPS1*, and the *4CLs*, *CNL*, and other *BEATs* co-expressed with *HmBEAT1*, are likely under the coordinated control of specific transcription factors, thereby amplifying the overall metabolic flux to influence VOC emission and, consequently, the sensory fragrance type. These hypotheses, however, require further validation through targeted transcriptional regulation experiments.

Our functional validation focused on the top-ranking candidates (*HcTPS1* and *HmBEAT1*) from the two key gene modules most strongly associated with fragrance variation. This targeted approach established clear gene-metabolite linkages for the characteristic volatiles (eucalyptol, benzyl acetate) of their respective scent types. The potential roles of other co-expressed genes within these modules, and their contributions to the full spectrum of *Hedychium* fragrance diversity, constitute important avenues for future research.

### Potential factors influencing the formation of sensory fragrance in *Hedychium*

It is important to emphasize that while this study identified and functionally characterized two key genes associated with *Hedychium* sensory fragrance through multi-dimensional data, numerous other factors likely influence scent formation. Variables such as temperature, light, humidity, endogenous hormones, developmental stage, transcriptional regulation, and post-transcriptional control can all affect the expression of key scent biosynthetic enzyme genes, their activity, and ultimately the emission of specific VOCs, potentially leading to changes in perceived fragrance [59–61]. Indeed, several studies have found that IAA [62], ABA [63], melatonin [64], and different developmental stages [34, 35, 43] significantly influence fragrance variation in *Hedychium*. Preliminary evidence also suggests that *HcMYBs* [65, 66], *HcARF5* [67], and microRNAs [68] can regulate the emission of key VOCs by directly or indirectly affecting the expression of *TPSs* and *BSMTs*. While these factors significantly impact VOC emission in *Hedychium*, whether they substantially alter the sensory fragrance category or completely transform the fragrance type remains an open question for deeper investigation. Furthermore, these regulatory studies have primarily focused on the single species *H. coronarium*, indicating a need for broader taxonomic sampling. The sensory evaluation wheel and the preliminary classification of typical fragrance types established in this study provide a scientific methodology to further investigate whether the influences of these multifactorial variables on VOCs translate to perceptible changes in sensory fragrance. Moreover, the chemical diversity underlying different sensory fragrances across species suggests that future research on *Hedychium* fragrance should place greater emphasis on studying multiple species representing distinct sensory types.

**Figure 7:**
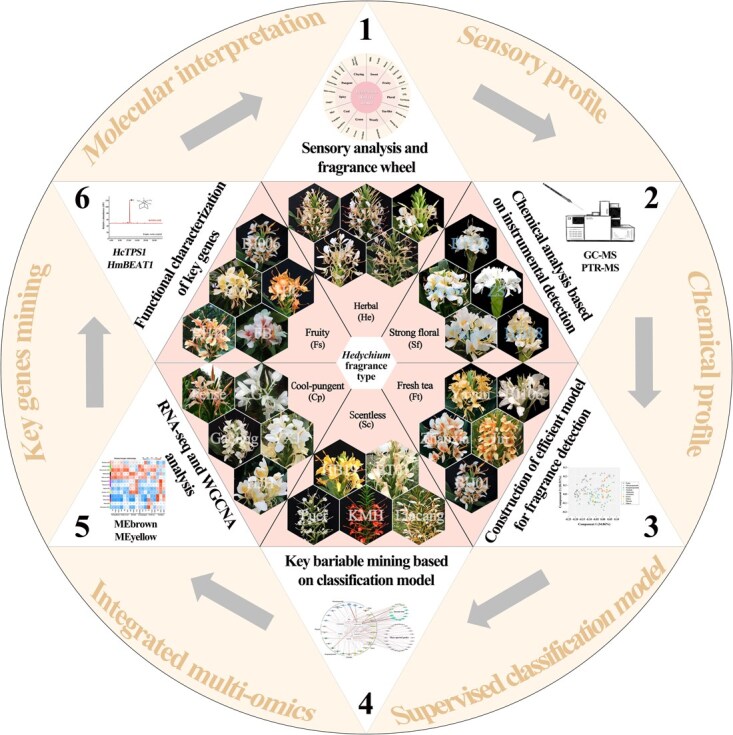
An integrated analytical framework for the mechanistic basis of sensory fragrance diversity in specific plants (*Hedychium*).

In summary, this study established an integrated analytical framework encompassing (Fig. 7): (i) sensory analysis and fragrance wheel development, (ii) comprehensive VOC profiling, (iii) construction of efficient model for floral scent detection; (iv) optimized machine learning models (e.g. supervised classification) for different fragrance types discrimination and mined the key variables, (v) integrated multi-omics, and (vi) molecular characterization of key biosynthetic genes. By connecting ‘key genes–floral volatiles–sensory attributes’, we provide a systematic approach for investigating fragrance diversity formation mechanisms in *Hedychium* flowers, offering a valuable paradigm for future research in floral scent biology. These findings provide a mechanistic explanation for fragrance diversification in *Hedychium* and demonstrate how sensory-driven omics can decipher complex traits in non-model ornamental species. The identified gene modules and enzymes also offer direct targets for molecular breeding programs aimed at fragrance enhancement, establishing a reproducible strategy for trait-directed improvement in ornamental plants.

## Materials and methods

### Plant materials

All plant materials were cultivated at the Flower Research Center of South China Agricultural University in Guangzhou, China (23.16° N, 113.36° E). For experimental analyses, cut flowers were collected prior to the opening of the first bloom in each inflorescence. The harvested flowers were immediately transferred to a controlled environment chamber for vase culture under standardized conditions (27°C ± 2°C, 75%–80% relative humidity). Following a 3-h stabilization period, fragrance characterization was initiated. Flowers at full bloom stage from various *Hedychium* cultivars were subsequently sampled for headspace solid-phase microextraction gas chromatography–mass spectrometry (HS–SPME–GC–MS) and proton transfer reaction time-of-flight mass spectrometry (PTR–ToF–MS) analyses. A minimum of three biological replicates were included for each analytical procedure.

### Panel composition, training, and consistency assessment

A sensory panel comprising ten trained evaluators (five males and five females, aged 21–30 years old) participated in this study. All panelists had over two years of specific experience in floral fragrance research and evaluation. Prior to formal assessments, each panelist’s olfactory function was verified using a standardized odor identification test to ensure normal odor sensitivity and discrimination ability [69]. The panel then completed a structured training program based on established principles for descriptive sensory analysis [70]. This included systematic exposure to a wide range of floral scent references, including *Hedychium* samples and representative fragrance standards, to calibrate descriptive terminology and intensity scaling. Panelists practiced using a 6-point intensity scale (0 = odorless to 5 = very strong) with physical reference anchors.

To ensure data reliability, intra-panelist reproducibility and inter-panelist consistency were quantitatively evaluated following recommended sensory-analysis guidelines [71]. Each panelist evaluated hidden duplicate samples of two reference cultivars during the same session. Only panelists who achieved ≥85% agreement in descriptor selection and intensity rating for these duplicates were retained for final profiling. Inter-panelist consistency was assessed using Cronbach's alpha for intensity scores of primary descriptors and Fleiss’ kappa for descriptor agreement [72]. Panelists received periodic feedback during training to align their evaluations with the group consensus. During formal evaluation, fresh flowers at full bloom were collected during peak volatile emission periods and placed in 250-ml glass bottles wrapped in aluminum foil to prevent visual bias. Samples were presented in individual sensory booths under controlled environmental conditions, following a randomized complete block design across multiple sessions to minimize order and carry-over effects.

### Construction of the sensory evaluation wheel and selection of representative materials

Twenty-five *Hedychium* cultivars exhibiting diverse fragrance characteristics were evaluated (Table S1). Fresh flowers at full bloom stage (~2 g) were collected during peak volatile emission periods (8:00–10:00 a.m.) and placed in 250-ml amber glass jars to prevent visual bias during evaluation. Each sample underwent comprehensive sensory assessment including odor descriptor identification and intensity grading on a standardized 6-point scale (0 = odorless, 1 = very weak, 2 = weak, 3 = moderate, 4 = strong, 5 = very strong).

The collected descriptors underwent systematic refinement through multiple stages. Initial screening eliminated redundant, quantitative, or irrelevant terms, followed by categorization based on published fragrance lexicons. The significance of sensory descriptors was quantified to consolidate the evaluation vocabulary. Following established practices for identifying key attributes in sensory profiling [73], the geometric mean (*M*) was calculated for each descriptor using the formula *M* = √[*F* (%) × *I* (%)], where *F* (%) represents the citation frequency (percentage of panelists using the term) and *I* (%) denotes the average perceived intensity (scored on a 0–5 scale) expressed as a percentage of the maximum possible intensity. This integrated metric accounts for both the commonality and perceptual strength of each odor characteristic. Descriptors with *M* > 0.05 were designated as primary descriptors. To ensure the sensory wheel captured the full olfactory spectrum, distinctive odor notes that were consistently mentioned by at least two panelists—regardless of their Mvalue—were also documented and included as supplementary descriptors. Subsequent hierarchical clustering analysis consolidated synonymous terms, generating a preliminary descriptor set that was further refined into the final sensory evaluation wheel.

Six distinct *Hedychium* fragrance types were delineated through this process, each represented by a characteristic cultivar: strong floral (*H. coronarium* ‘ZS’), fruity (*H. flavescens* ‘EM’), herbal (*H. menghaiense* ‘MH’), cool-pungent (*H. forrestii* ‘Gaoling’), fresh tea (*H.* ‘Jin’), and scentless (*H. coccineum* ‘KMH’). For each fragrance type, four additional cultivars exhibiting similar olfactory profiles were selected, yielding a total of thirty representative samples (Table S1). These carefully selected materials were subsequently subjected to HS–SPME–GC–MS and PTR–ToF–MS analyses to establish robust correlations between sensory attributes and VOC profiles.

### Volatile organic compounds detection and analysis by HS–SPME–GC–MS

#### Detection of VOCs using HS–SPME–GC–MS

The floral volatile profiling was conducted using HS–SPME–GC–MS, adopting a modified version of previously published methods [43]. For each analysis, freshly collected *Hedychium* flowers were transferred to 250 ml glass vials preloaded with 1.728 μg of ethyl decanoate serving as the internal standard. The vials were promptly sealed with tin foil to prevent volatile loss and allowed to equilibrate for 15 min at 26°C ± 2°C. Volatile compounds were then adsorbed onto a PDMS/DVB/CAR (50/30 μm) SPME fiber for 15 min under the same temperature conditions. The analytical system comprised an Agilent 7890A gas chromatograph interfaced with an Agilent 5975C mass spectrometer (single quadrupole detector). Separation was carried out on a DB-5MS capillary column (30 m length × 0.25 mm internal diameter) using ultra-pure helium as carrier gas at a constant flow rate of 1 ml∙min^−1^. The oven temperature program was initiated at 40°C (3 min hold), then increased at 5°C∙min^−1^ to a final temperature of 250°C. The mass spectrometer operated in electron impact ionization mode (70 eV) with the transfer line and ion source temperatures set at 280°C and 170°C, respectively. The complete analytical run required 28 min per sample. To ensure analytical reliability, each sample was analyzed with three to five biological replicates.

#### Identification of VOCs

VOCs were identified through comprehensive spectral matching against three reference sources: the NIST08 mass spectral library, published literature, and authentic standards. Mass spectra were acquired in scan mode (20–500 amu) and processed using Mass Hunter Qualitative Analysis Workflow software, with compounds retained only when exhibiting >80% similarity to library entries. Linear Retention Indices (LRIs) were calculated using n-alkane standards (C7–C40) under identical chromatographic conditions. Compound identities were confirmed when the calculated LRI (LRI_CAL_) matched the reference LRI (LRI_NIST_) within ±20 units [74].

#### Quantitative analysis of VOCs

Two normalization approaches were employed for VOC quantification [23]: (i) internal standard normalization (‘GC’ data) and (ii) total sum normalization (‘GCP’ data). Additionally, VOCs were classified into 10 primary incense tone (pungent, spicy, herbal, cool, green, fruity, floral, sweet, woody, and others) based on their sensory attributes documented in FlavorDB2 [33]. The relative abundances of these incense tone were calculated as both absolute values (‘IT’ data) and proportions (‘ITP’ data), providing complementary perspectives on fragrance composition.

### Analysis and quantification of VOCs by PTR–ToF–MS

VOCs were also analyzed using proton transfer reaction time-of-flight mass spectrometry (PTR–ToF–MS 1000, Ionicon Analytik GmbH, Austria) according to our previously established methods [34, 43]. The experimental setup incorporated an integrated system consisting of an inflation pump, carbon rod filter, glass rotameter, and 250-ml sample vials, with all components connected using polytetrafluoroethylene tubing to ensure minimal VOC adsorption during analysis. The PTR–ToF–MS instrument was operated under optimized conditions with the drift tube maintained at 2.30 mbar pressure and 80°C temperature, applying 600 V voltage to achieve an E/N ratio of approximately 137 Td (Townsend). For each sample, real-time VOC measurements were performed through direct headspace sampling at a flow rate of 50 sccm, while zero air was continuously introduced at 1 l∙min^−1^ to maintain system pressure stability. Ambient air was pre-filtered through a carbon rod purification system before entering the analytical system. Mass spectral data acquisition covered the *m*/*z* range of 20.0 to 245.0 atomic mass units with high resolution, with each sample analyzed for 30 to 60 s corresponding to 30 to 60 measurement cycles. To ensure data quality, systematic background correction was performed through blank measurements between samples, complemented by 1-min intervals to eliminate potential memory effects between consecutive analyses. Data collection and processing were conducted using dedicated software platforms, with raw data acquired through IoniTOF v.2.4.40 and subsequently processed using PTR–MS Viewer v.3.2.3.0. Preliminary VOC identification was achieved by matching measured mass-to-charge ratios against established references in the literature. Quantitative analysis was performed by calculating absolute headspace VOC concentrations from peak intensities based on the calibration methodology developed by Cappellin *et al.* [75], with the resulting comprehensive dataset designated as ‘PTR’ for further statistical and comparative analyses.

### RNA-Seq analysis

Based on the floral fragrance analysis results, we selected 18 accessions representing six distinct fragrance types (three biological replicates per accessions) for RNA-seq analysis. Flower petals collected at full bloom stage were immediately frozen in liquid nitrogen and stored at −80°C for subsequent RNA extraction. Total RNA was extracted using TRIzol® Reagent (Invitrogen) following the manufacturer's protocol, with genomic DNA removed using DNase I (TaKara). RNA quality was assessed using an Agilent 2100 Bioanalyzer and quantified with a NanoDrop ND-2000 spectrophotometer. Only high-quality RNA samples were used for library preparation.

RNA-seq libraries were prepared using the Illumina TruSeq™ RNA Sample Preparation Kit (San Diego, CA) with 1 μg of total RNA as input. Briefly, mRNA was isolated through polyA selection using oligo (dT) beads and fragmented. Subsequent steps included cDNA synthesis, end repair, A-tailing, and Illumina adapter ligation performed according to the manufacturer's protocol. The libraries were size-selected for 200 to 300 bp fragments using 2% Low Range Ultra Agarose gel electrophoresis, followed by 15 cycles of PCR amplification with Phusion DNA polymerase (NEB). After quantification using TBS380, the paired-end libraries were sequenced (150 bp × 2) on an Illumina NovaSeq 6000 platform (Shanghai BIOZERON Co., Ltd).

Raw sequencing reads were processed using Trimmomatic (version 0.36, http://www.usadellab.org/cms/uploads/supplementary/Trimmomatic) with parameters (SLIDINGWINDOW:4:15 MINLEN:75) for quality control and adapter trimming. The clean reads were then aligned to the *Hedychium coronarium* ‘ZS’ reference genome in orientation-aware mode using HISAT2 (https://ccb.jhu.edu/software/hisat2/index.shtml) with default parameters. Alignment quality was assessed using Qualimap v2.2.1 (http://qualimap.bioinfo.cipf.es/), and read counts per gene were generated using HTSeq (https://htseq.readthedocs.io/en/release_0.11.1/).

Differential gene expression analysis was performed using the edgeR package (http://www.bioconductor.org/packages/release/bioc/html/edgeR.html) in R. Gene expression levels were quantified as transcripts per million (TPM). Differentially expressed genes (DEGs) were identified using thresholds of |log2 fold change| > 2 and false discovery rate (FDR) < 0.05. Functional enrichment analysis of DEGs was conducted for Gene Ontology (GO) terms using Goatools (https://github.com/tanghaibao/Goatools) and KEGG pathways using KOBAS (http://kobas.cbi.pku.edu.cn/home.do), with statistical significance determined by Bonferroni-corrected *P*-value < 0.05.

### Cloning and sequence analysis of floral fragrance-related enzyme genes

Total RNA was extracted from flowers of *H. coronarium* ‘ZS’ and *H. menghaiense* ‘MH’ using RNAiso Plus reagent (TaKaRa) following the manufacturer’s protocol. Full-length cDNA sequences were amplified using KOD Plus high-fidelity DNA polymerase (TOYOBO). For phylogenetic analysis, amino acid sequences were aligned using ClustalX and rooted neighbor-joining trees were constructed with MEGA7 [76]. The species and accession numbers corresponding to the sequences used in the phylogenetic analysis are provided in Table S20.

### Heterologous expression of *HcTPS1* and *HmBEAT1* in *E. coli*

The coding sequences of HcTPS1 and HmBEAT1 were amplified from *H. coronarium* ‘ZS’ and *H. menghaiense* ‘MH’ flowers, respectively, using KOD-Plus DNA polymerase (TOYOBO) and subsequently cloned into the pET30a vector (Novagen) via EcoRI and NotI restriction sites. After sequence verification, recombinant plasmids were transformed into *E. coli* Rosetta (DE3) competent cells (Invitrogen). Protein expression was induced with isopropyl-β-d-thiogalactopyranoside (IPTG), followed by partial purification using Ni-NTA His·Bind Resins (Novagen) as previously described [35]. The empty pET30a vector served as negative control.

### Enzymatic characterization of recombinant HcTPS1 and HmBEAT1

Enzyme assays were conducted in 5 ml sealed glass vials to verify the activities of HcTPS1 and HmBEAT1. For HcTPS1, standard reactions (1 ml total volume) contained assay buffer (20 mM MgCl₂, 30 mM HEPES pH 7.5, 5 mM DTT), 20 μM geranyl diphosphate (GPP) substrate, and purified recombinant protein. HmBEAT1 reactions (200 μl total volume) consisted of 40 μl buffer (250 mM Tris–HCl pH 7.5, 25 mM MgCl₂, 25 mM KCl, 10 mM β-mercaptoethanol), 5 μl 6 mM acetyl-CoA, 2 μl 2 M benzyl alcohol, and 30 μl recombinant protein. After incubation at 30°C for 60 min, volatile products were collected using a PDMS/DVB/CAR (50/30 μm) SPME fiber (Supelco) and analyzed by GC–MS (Agilent 7890A GC system coupled with 5975C MSD). The instrumental analysis conditions and compound identification methods were consistent with the previously described GC–MS protocol for Hedychiumfloral volatile analysis.

### Transient expression of *HcTPS1* and *HmBEAT1* in planta

The open reading frames (ORFs) of EGFP (negative control), HcTPS1, and HmBEAT1 were amplified using KOD-Plus high-fidelity DNA polymerase (TOYOBO) with gene-specific primers and subsequently subcloned into the pGreenII 62-SK binary vector [77] via the SacI and KpnI restriction sites. The resulting constructs were transformed into *Agrobacterium tumefaciens* strain GV3101 (pSoup) competent cells. For agroinfiltration, bacterial cultures were resuspended in infiltration buffer (10 mM MES, pH 5.2, 10 mM MgCl₂, and 0.1 mM acetosyringone) to a final OD_600_ of 0.4. After incubation for 3 h at room temperature, the bacterial suspension was infiltrated into the abaxial side of leaves from 4-week-old *N. benthamiana* plants using a needleless syringe. Three days post-infiltration, 1 mM benzyl alcohol (BA) or salicylic acid (SA, pH 7.0) was infiltrated into the same leaf areas using the same method. The entire plant was then enclosed in a 500-ml glass container for headspace volatile collection. Following a 12-h collection period, VOCs were analyzed by HS–SPME–GC–MS using the same analytical method described for the recombinant enzyme assays.

### Statistical analysis

All statistical analyses were performed in R unless otherwise specified. Spearman correlation, hierarchical clustering, and PCA were conducted using built-in R functions. PLS regression and Jackknife tests were implemented using the ‘pls’ package [78]. PLS-discriminant analysis (PLS-DA), VIP scores, and regression coefficients were calculated using the ‘ropls’ package (https://bioconductor.org/packages/release/bioc/html/ropls.html). For PLS-DA modeling, fragrance types served as Y variables (dependent variables) while 31 orthogonal datasets (derived from GC, GCP, IT, ITP, and PTR data) constituted X variables (independent variables). Correlation networks were visualized using Cytoscape 3.10.1 [79]. Weighted gene co-expression network analysis (WGCNA) of differentially expressed genes was performed using the ‘WGCNA’ package [80].

## Supplementary Material

Web_Material_uhag063

## Data Availability

The nucleotide sequences of *HcTPS1* (PX095165) and *HmBEAT1* (PX095164) have been deposited in GenBank. The raw transcriptome data have been submitted to the NCBI Sequence Read Archive (SRA) database (PRJNA1303972). All other data are available from the corresponding author on reasonable request.
