# Supplementary material for: From fragrance wheel to functional genes: a multi-omics investigation into fragrance type formation in ornamental Hedychium flowers
Source: Hortic Res. 2026 Feb 27;13(6):uhag063. doi: 10.1093/hr/uhag063 (PMC13253351; doi:10.1093/hr/uhag063)
Supplement: Web_Material_uhag063 [file web_material_uhag063.zip › Supplementary Figures-R1.pdf]

Supplementary Figures

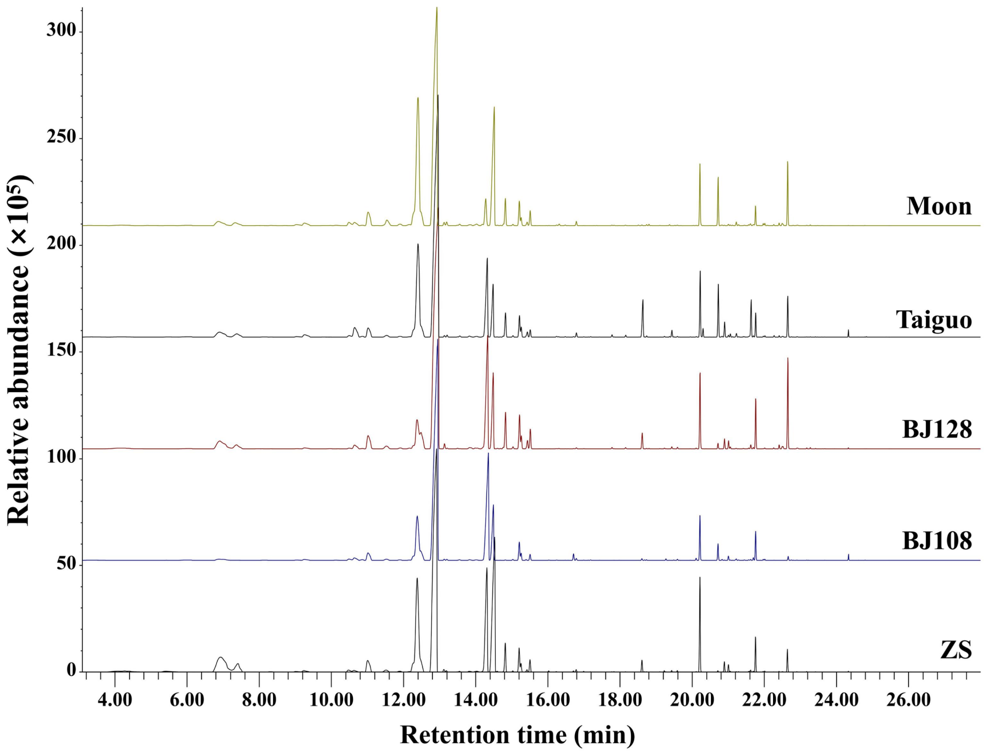

Fig. S1 Representative total ion chromatograms (TICs) of five *Hedychium* samples with strong floral aroma based on HS–SPME–GC–MS.

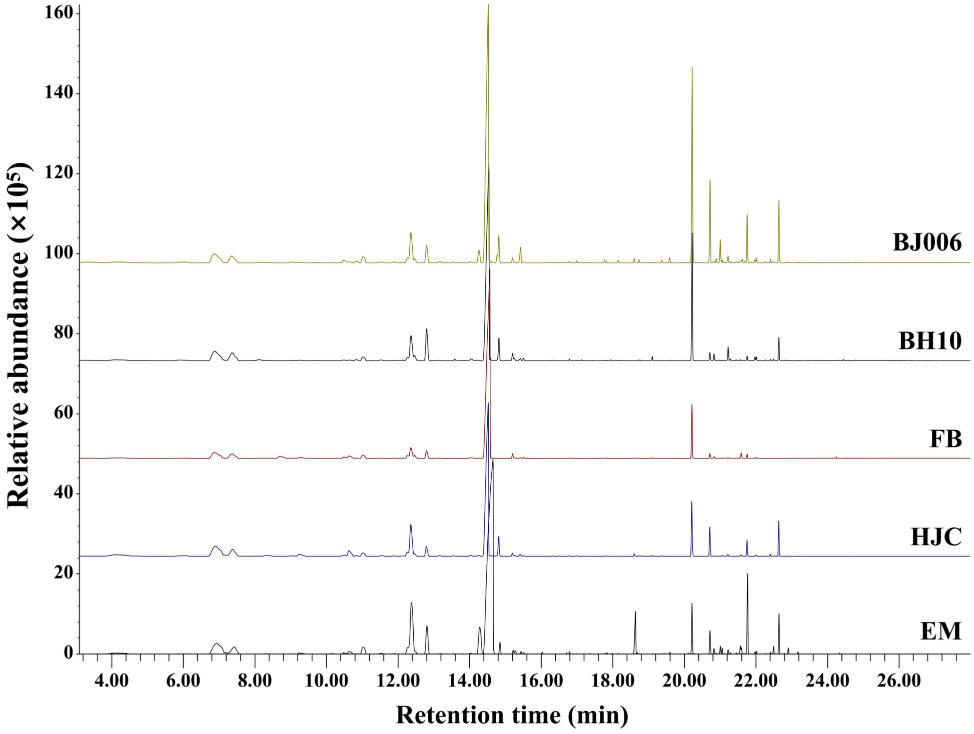

Fig. S2 Representative TICs of five *Hedychium* samples with fruity aroma based on HS–SPME–GC–MS.

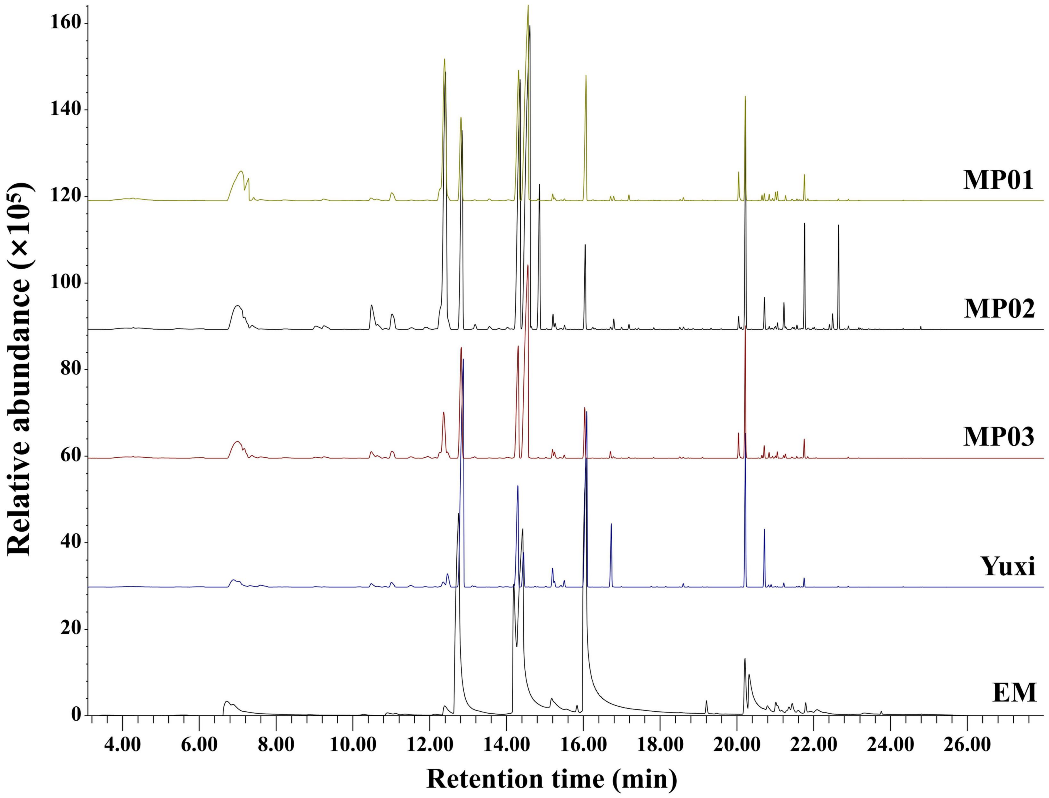

Fig. S3. Representative TICs of five *Hedychium* samples with herbal aroma based on HS–SPME–GC–MS.

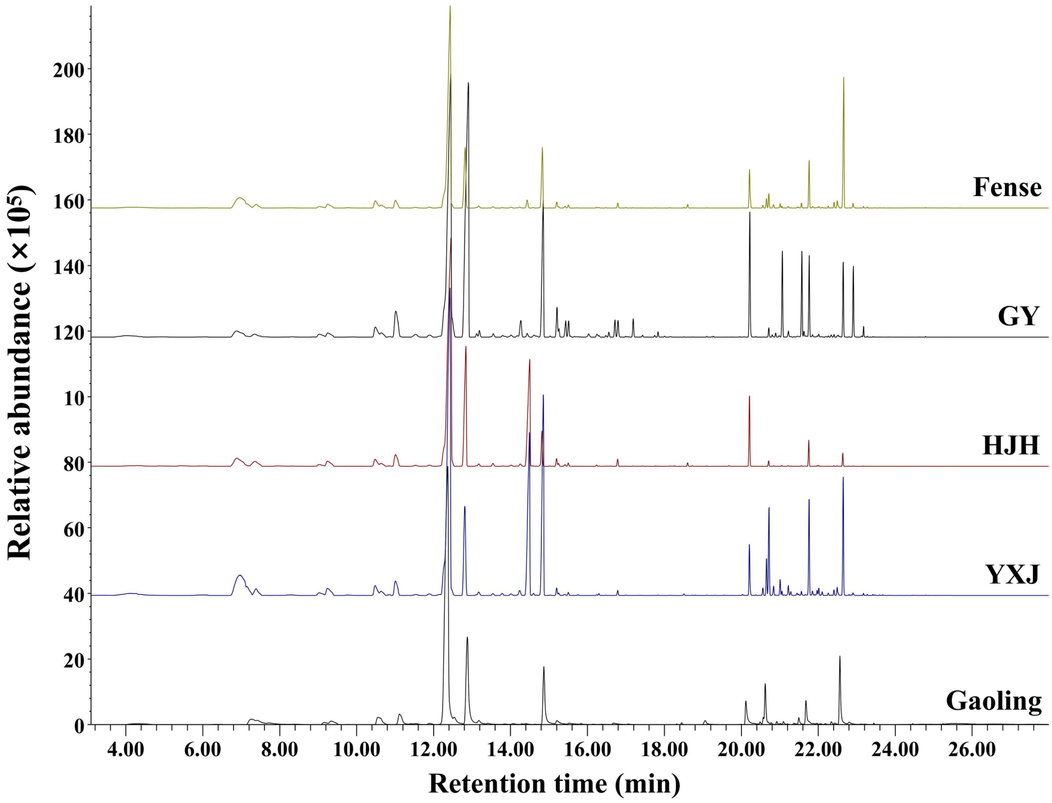

Fig. S4. Representative TICs of five *Hedychium* samples with cool-pungent aroma based on HS–SPME–GC–MS.

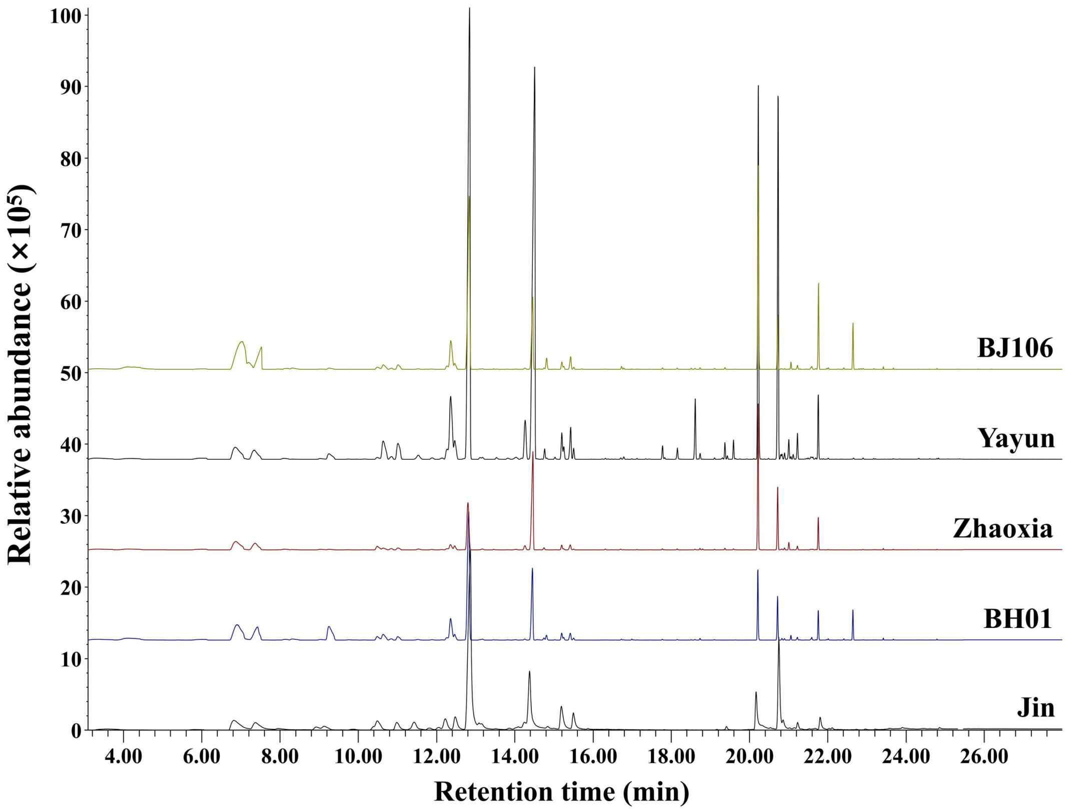

Fig. S5. Representative TICs of five *Hedychium* samples with fresh tea aroma based on HS–SPME–GC–MS.

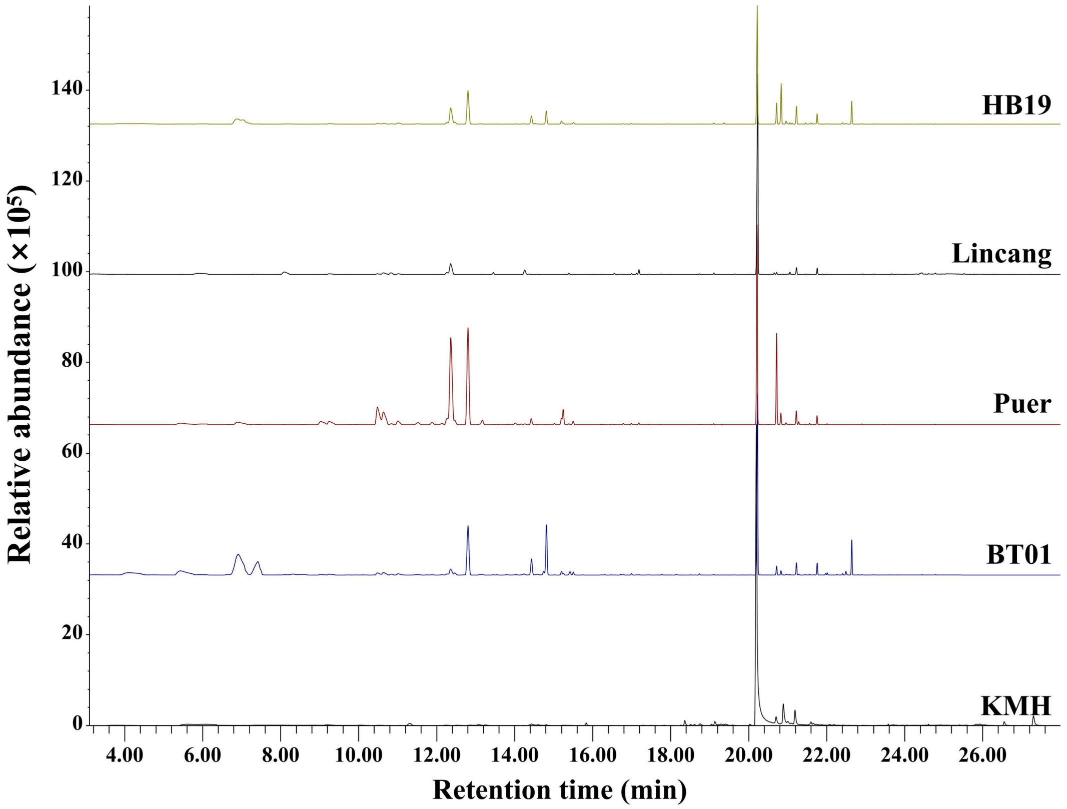

Fig. S6. Representative TICs of five *Hedychium* samples classified as scentless based on HS–SPME–GC–MS.

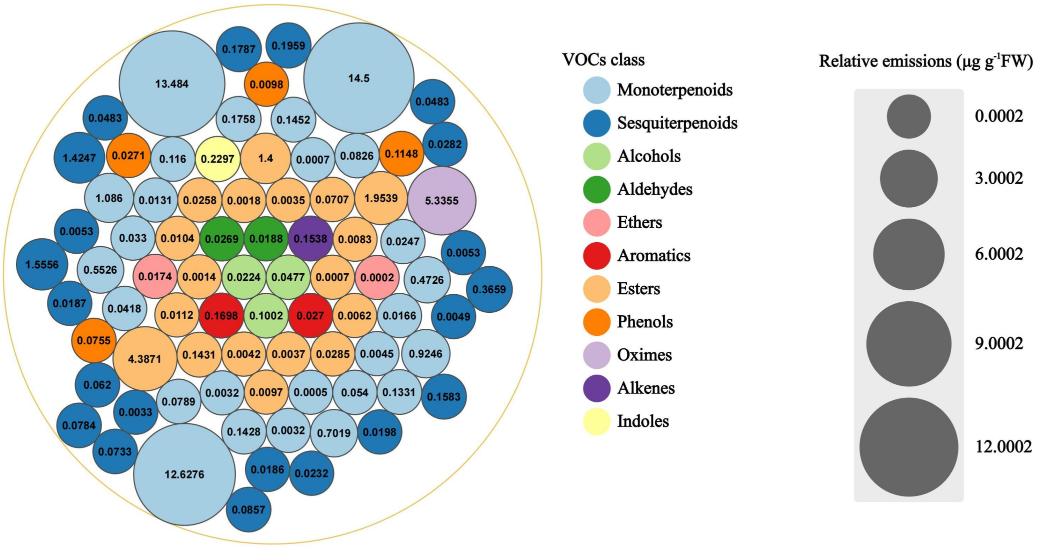

Fig. S7. Counts of 81 VOCs identified from 30 *Hedychium* samples via HS–SPME–GC–MS.

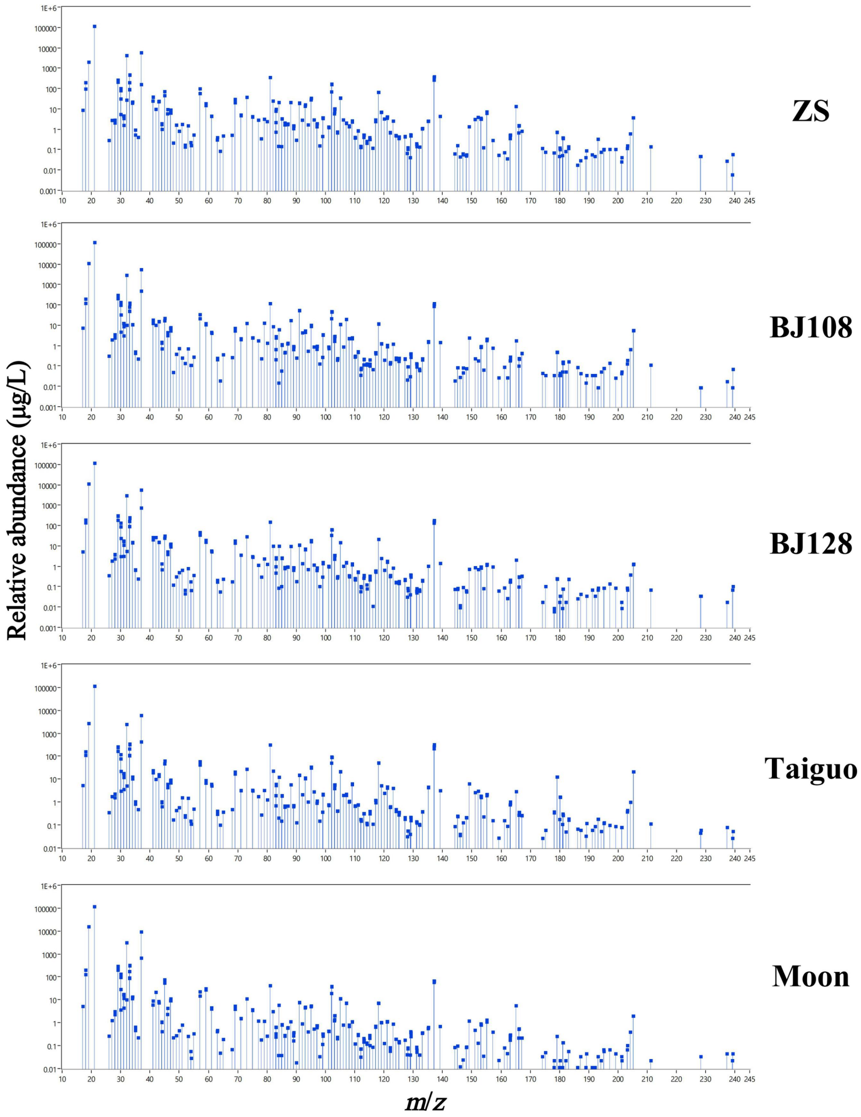

Fig. S8. Relative abundance profiles of representative mass spectral peaks for five *Hedychium* samples with strong floral aroma (PTR–ToF–MS).

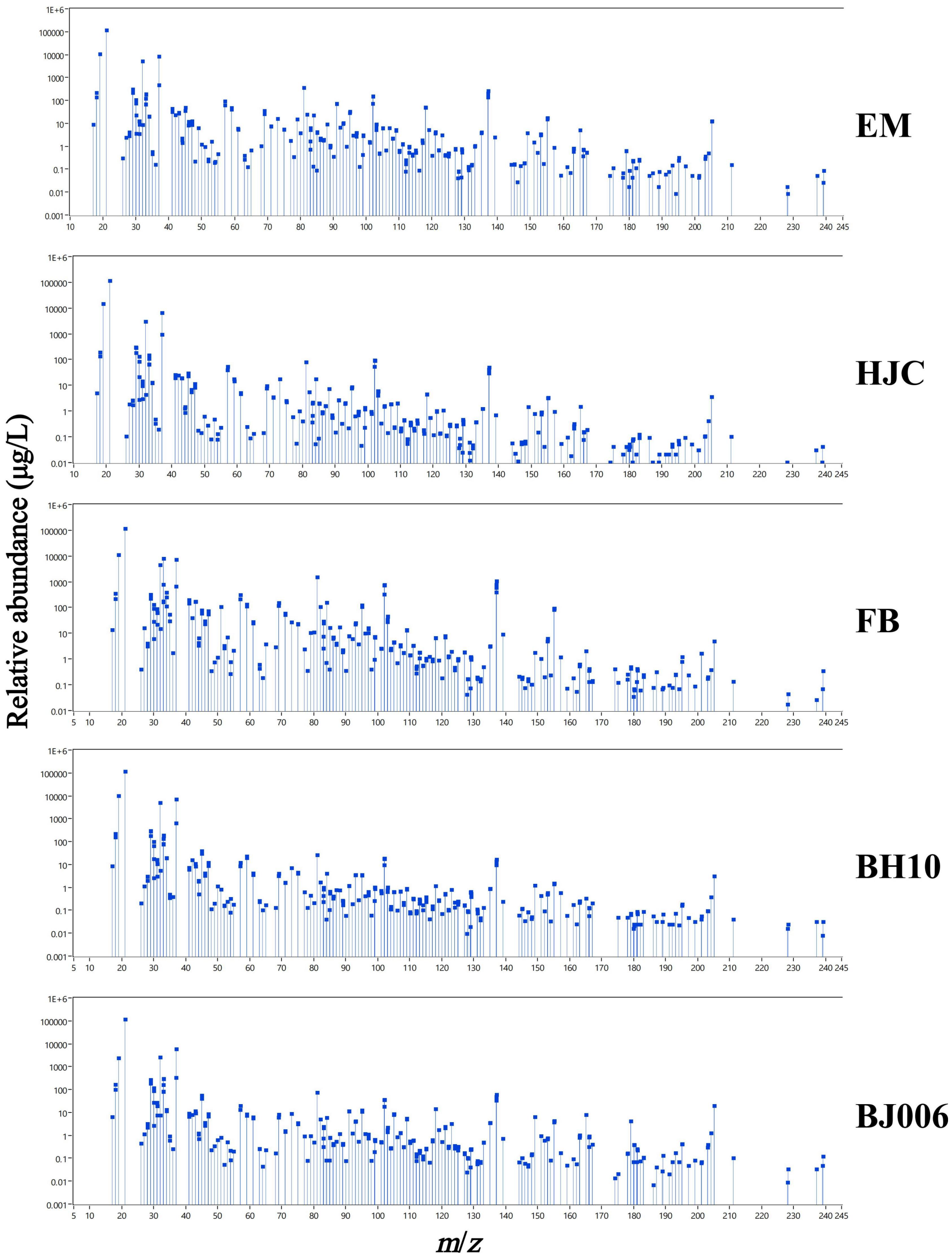

Fig. S9. Relative abundance profiles of representative mass spectral peaks for five *Hedychium* samples with fruity aroma (PTR–ToF–MS).

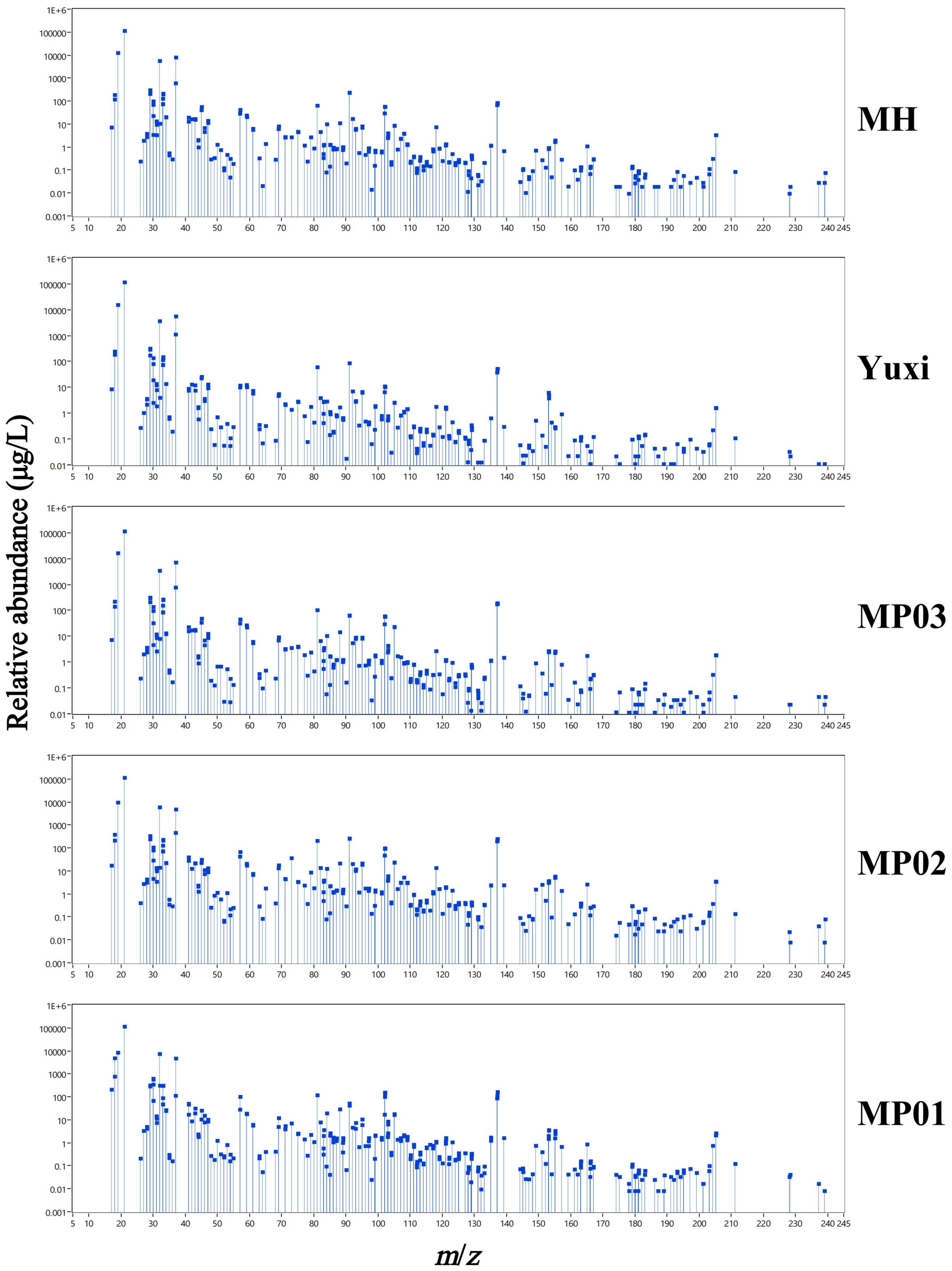

Fig. S10. Relative abundance profiles of representative mass spectral peaks for five *Hedychium* samples with spicy aroma (PTR–ToF–MS).

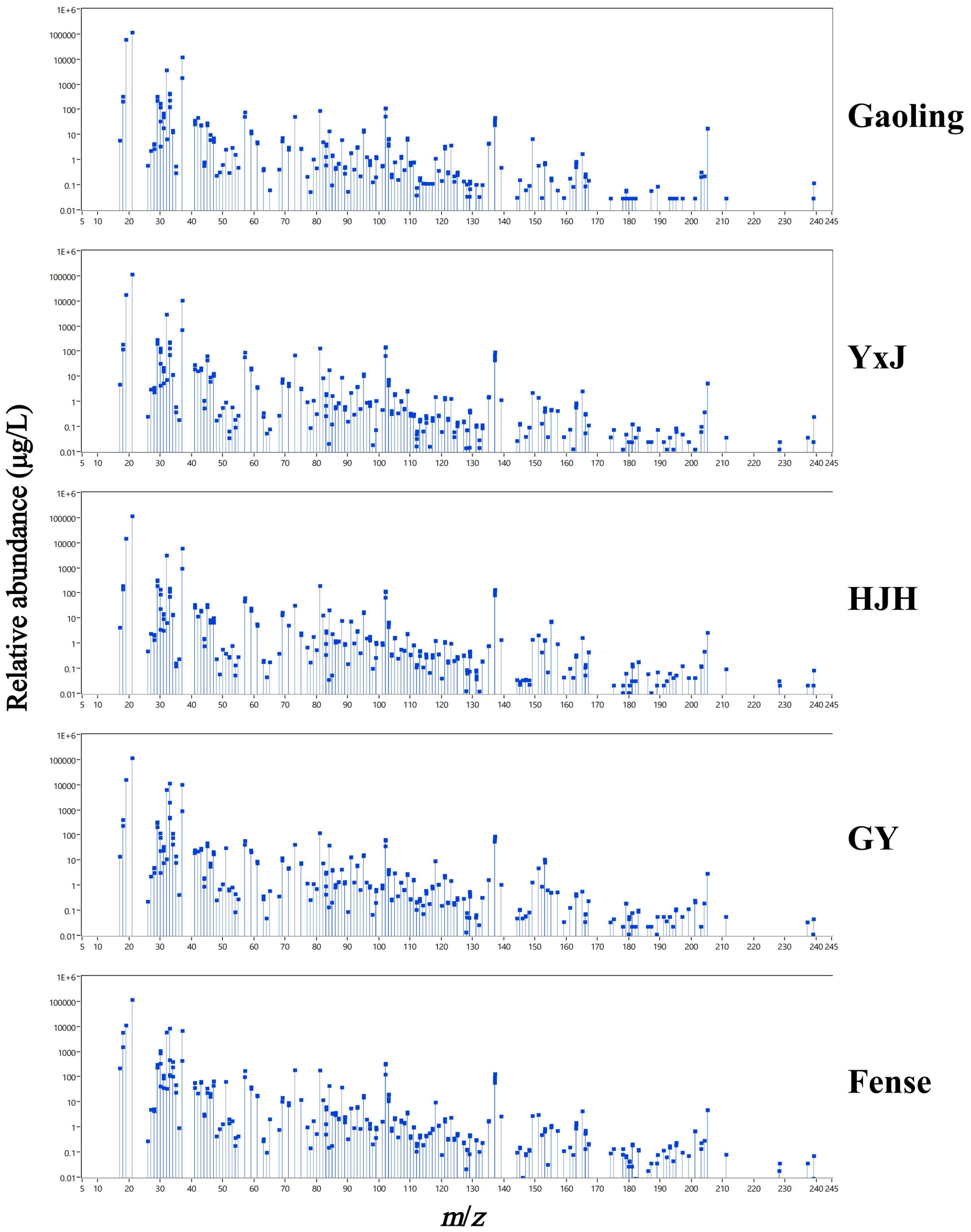

Fig. S11. Relative abundance profiles of representative mass spectral peaks for five *Hedychium* samples with cool-pungent aroma (PTR–ToF–MS).

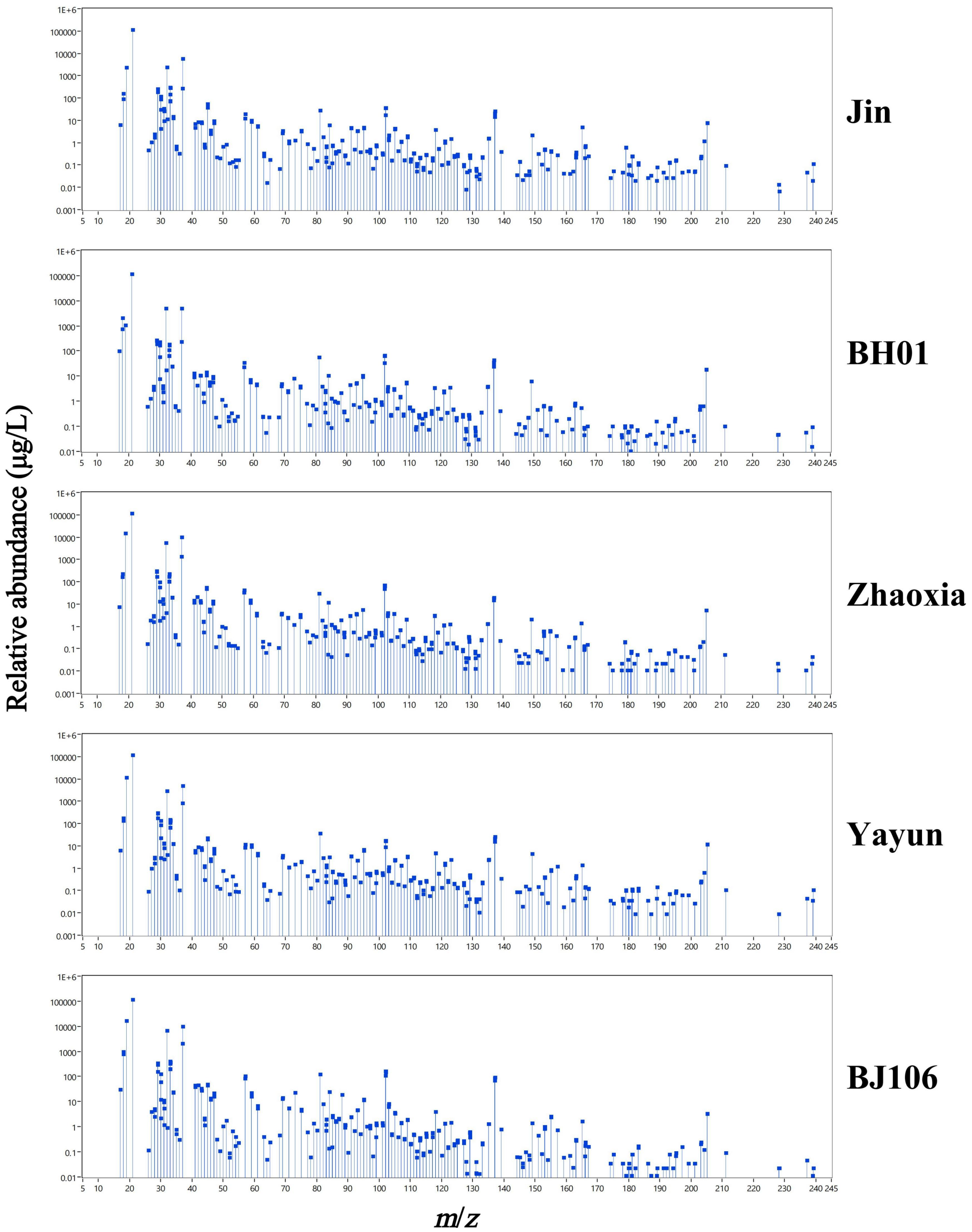

Fig. S12. Relative abundance profiles of representative mass spectral peaks for five *Hedychium* samples with fresh tea aroma (PTR–ToF–MS).

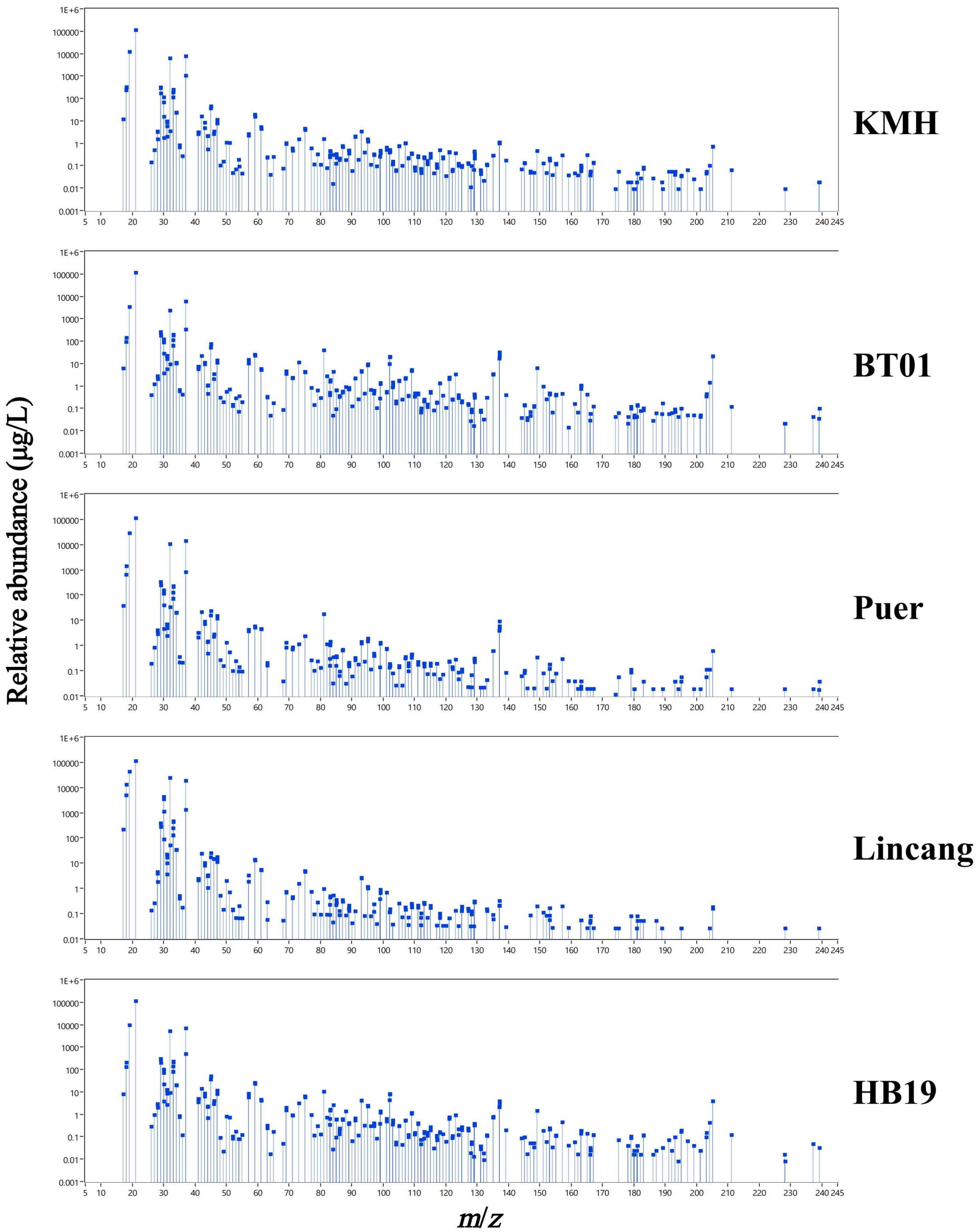

Fig. S13. Relative abundance profiles of representative mass spectral peaks for five *Hedychium* samples classified as scentless (PTR–ToF–MS).

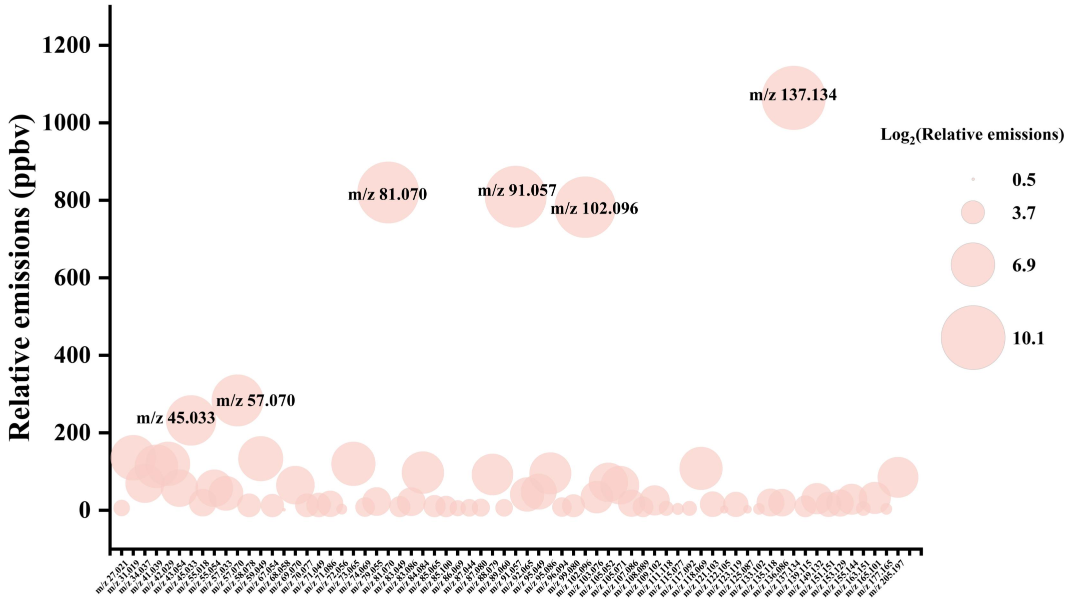

Fig. S14. Comparative analysis of 68 mass spectral peaks and their relative emissions identified via PTR–ToF–MS.

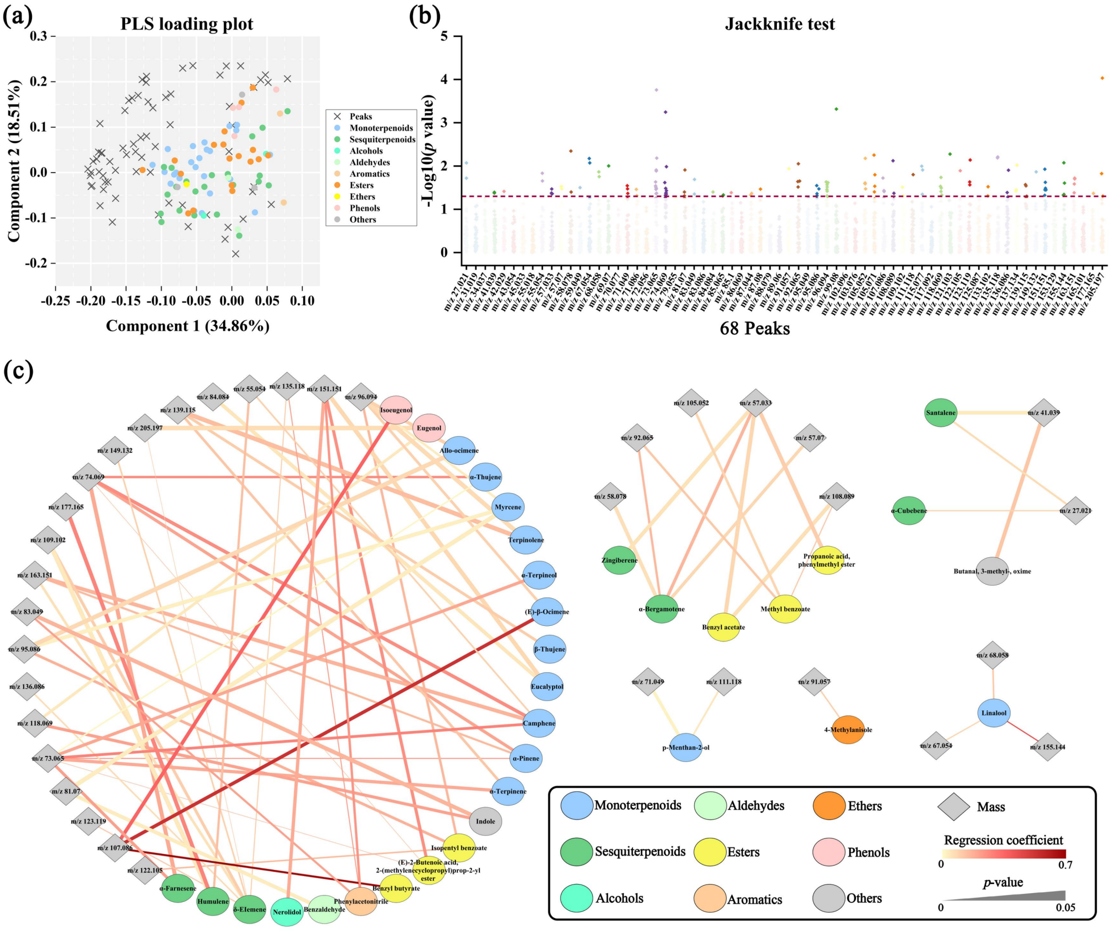

Fig. S15. Association analysis between HS–SPME–GC–MS and PTR–ToF–MS data using PLS regression. (a) PLS loading plot. (b) Jackknife test (red line: P = 0.05; colored points above the line indicate significantly associated VOCs). (c) Correlation network between significantly associated VOCs and mass spectral peaks.

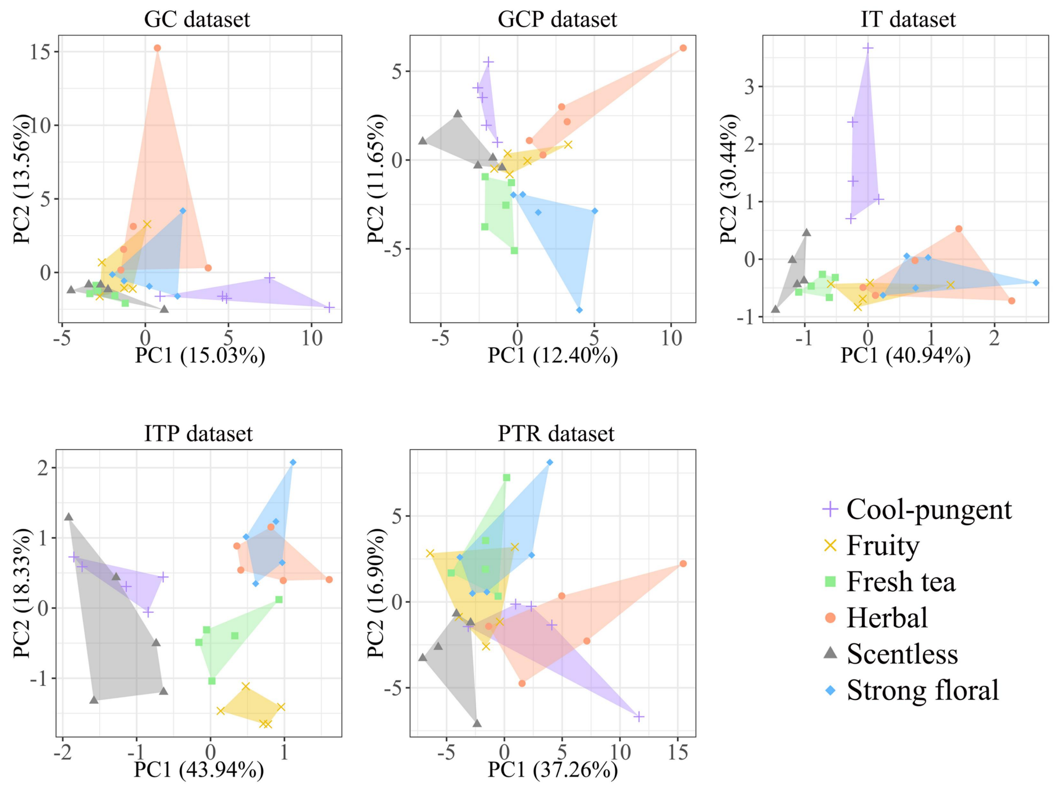

Fig. S16. PCA score plots of five raw datasets.

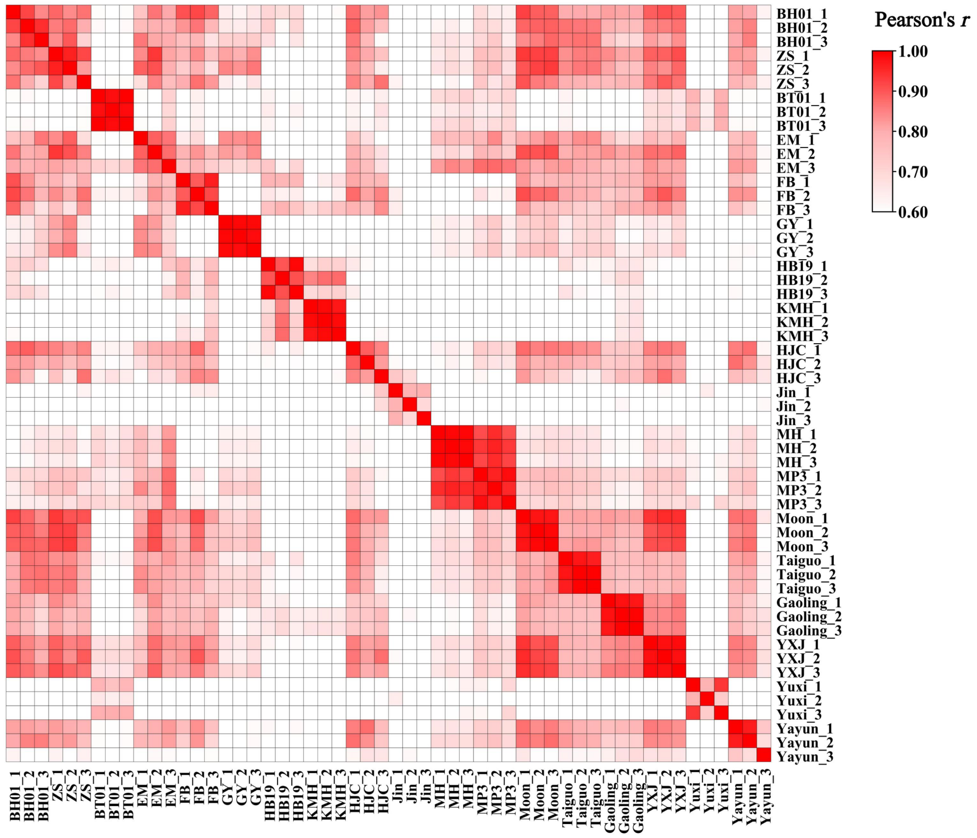

Fig. S17. Correlation heatmap of 54 floral samples from 18 representative *Hedychium* varieties across six aroma types.

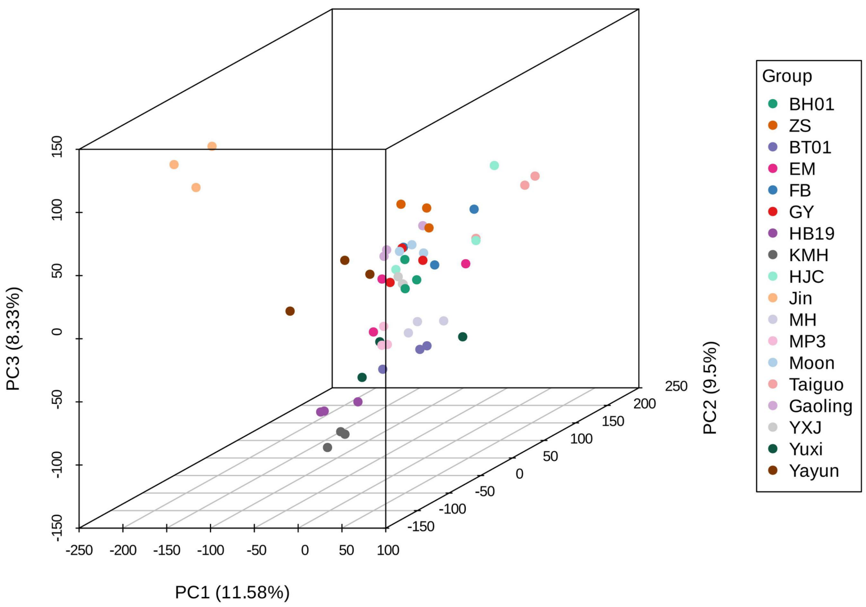

Fig. S18. PCA plot of 54 floral samples from 18 representative *Hedychium* accessions across six aroma types.

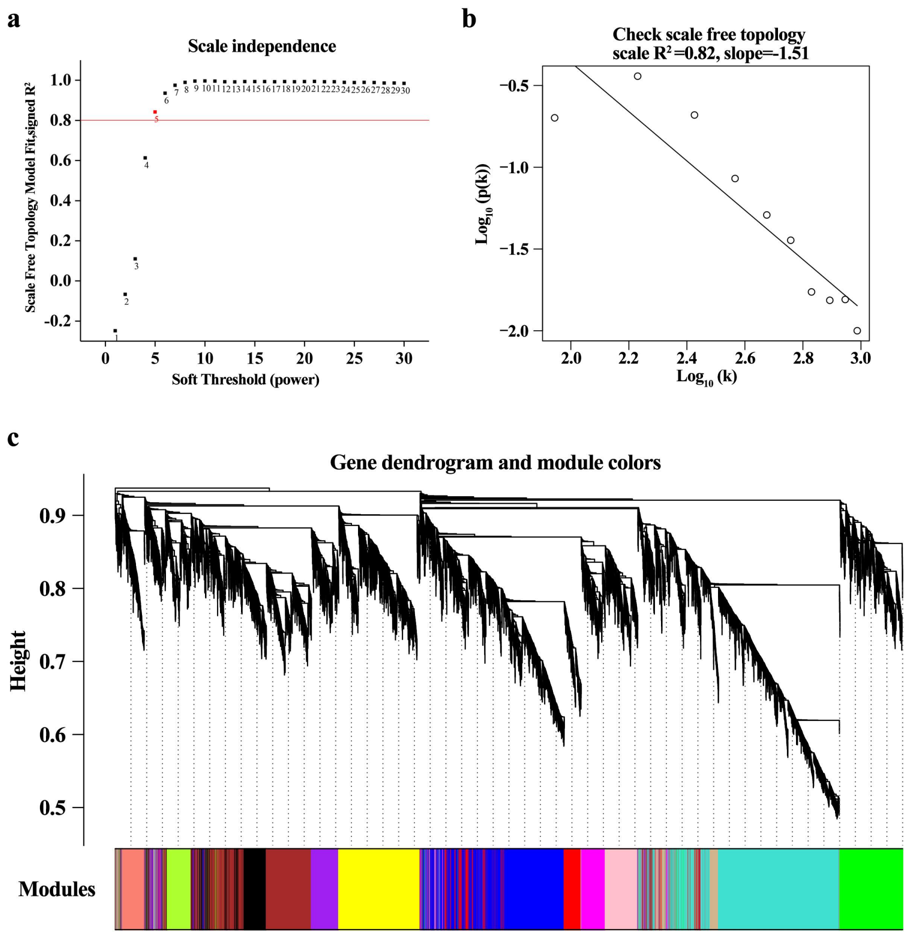

Fig. S19. Weighted gene co-expression network analysis (WGCNA) of transcriptomic data from 18 *Hedychium* accessions representing six aroma types. (a) Soft threshold selection. (b) Scale-free topology fit analysis at soft threshold = 5. (c) Co-expressed gene module clustering.

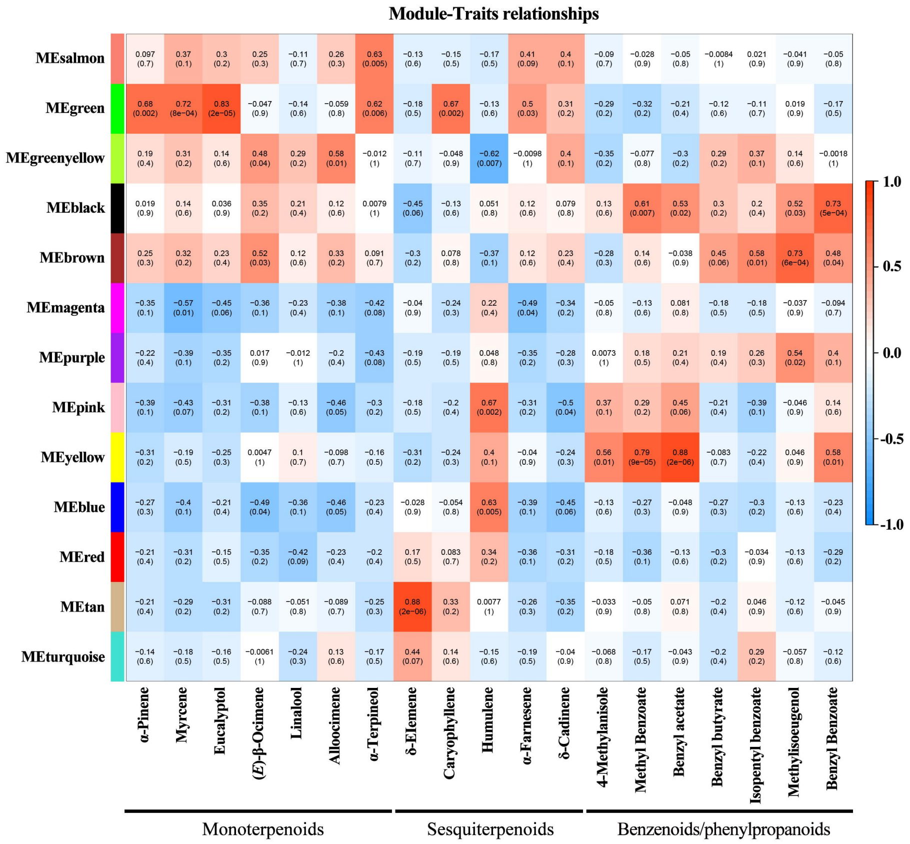

Fig. S20. Association analysis between major floral VOCs and 13 gene modules via WGCNA.

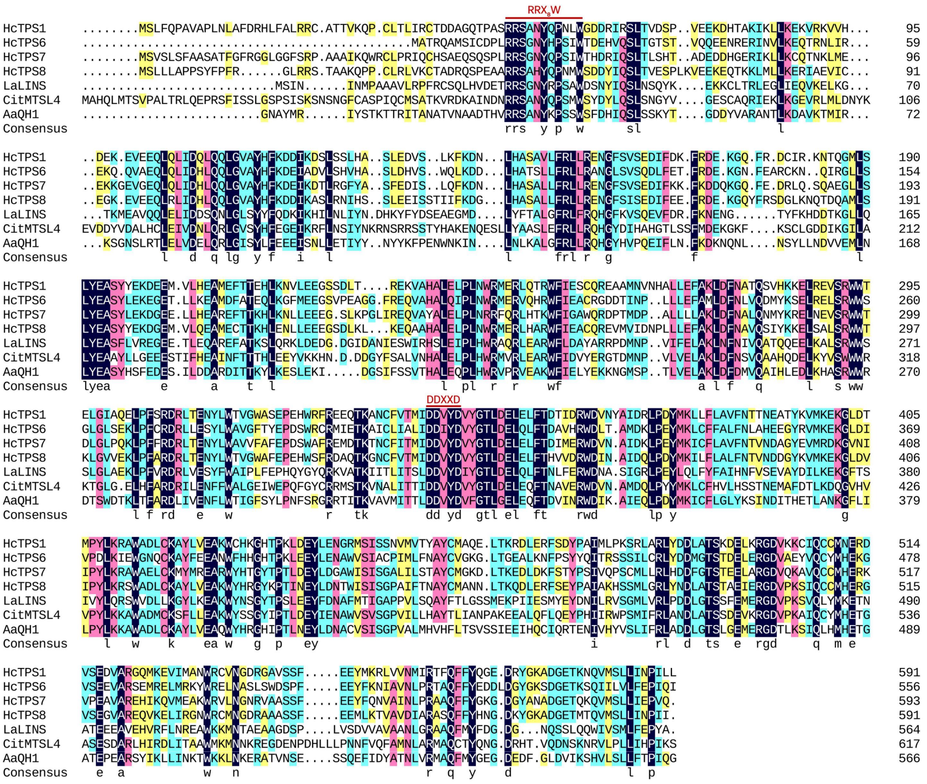

Fig. S21. Amino acid sequence alignment of HcTPS1 with terpene synthases from other plant species.

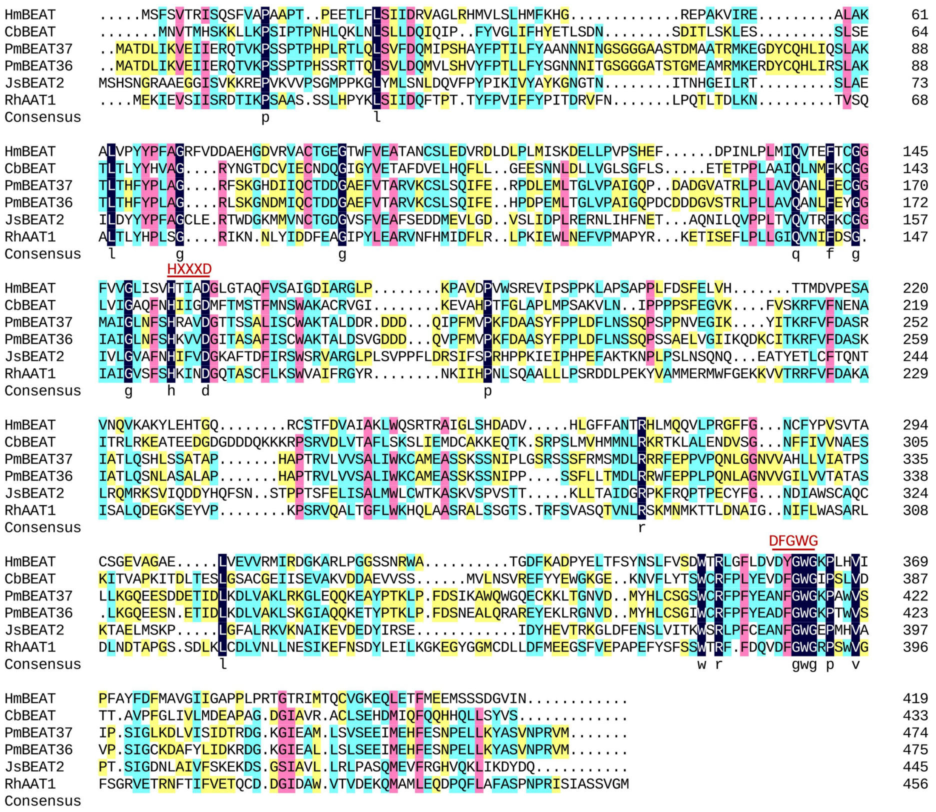

Fig. S22. Amino acid sequence alignment of HmBEAT1 with BAHD acyltransferase family enzymes from other plant species.
